# Abdominal Acupuncture as an Adjunctive Therapy for the Recovery of Motor Function After Stroke: A Systematic Review and Meta-Analysis of Randomized Controlled Trials

**DOI:** 10.3389/fneur.2021.705771

**Published:** 2021-09-28

**Authors:** Jie Zhan, Buhui Xiong, Peiming Zhang, Yiqiao Wang, Yuyuan Tang, Lechang Zhan, Liming Lu

**Affiliations:** ^1^Postdoctoral Research Station, Guangdong Provincial Hospital of Chinese Medicine, The Second Affiliated Hospital of Guangzhou University of Chinese Medicine, Guangzhou, China; ^2^Department of Rehabilitation, Guangdong Provincial Hospital of Chinese Medicine, Guangzhou, China; ^3^Clinical Research and Data Center, South China Research Center for Acupuncture and Moxibustion, Medical College of Acu-Moxi and Rehabilitation, Guangzhou University of Chinese Medicine, Guangzhou, China

**Keywords:** abdominal acupuncture, stroke, motor function, systematic review, alternative and complementary medicine

## Abstract

**Background:** Bo's abdominal acupuncture (BAA) is a novel therapy in alternative and complementary medicine and has been frequently used for stroke recovery in recent decades. However, no systematic evidence has been performed to confirm the effect and safety of BAA as an adjunctive therapy for post-stroke motor dysfunction (PSMD).

**Objectives:** This review aimed to assess the efficacy and safety of BAA as an adjunctive therapy for improving allover motor function, upper limb motor function, lower limb motor function, and activities of daily living (ADL) in patients with PSMD.

**Methods:** Seven databases were searched from inception to December 2020: Embase, PubMed, Cochrane Library, Chinese Biological Medicine Database, Chinese Scientific Journal Database, WAN FANG, and the China National Knowledge Infrastructure. All randomized controlled trials (RCTs) involving BAA plus another therapy vs. the same other therapy alone were identified. The methodological quality of the included trials was assessed according to the Cochrane risk of bias criteria. If more than half of the domains in a study are at low risk of bias, the overall quality of the study is low risk. We conducted a meta-analysis for primary outcomes using a random effects model and performed a narrative summary for the secondary outcome. We also conducted subgroup analysis for primary outcomes based on different add-on treatments to BAA. Random effects and fixed effects models were used to test the robustness of the pooled data. We also tested the robustness of the meta-analysis using specific methodological variables that could affect primary outcome measures.

**Results:** *Twenty-one* trials with 1,473 patients were included in this systematic review. The overall quality of the 14 included trials (66.7%) was low risk. Meta-analyses indicated that the effect of the BAA group was better than that of the non-EA group on the Fugl-Meyer Assessment Scale (FMA) (weight mean difference (WMD) 9.53, 95% confidence interval (CI) 7.23 to 11.83, *P* < 0.00001), FMA for upper extremities (WMD 11.08, 95% CI 5.83 to 16.32, *P* < 0.0001), FMA for lower extremities (WMD 5.57, 95% CI 2.61 to 8.54, *P* = 0.0002), and modified Barthel Index (standardized mean difference (SMD) 1.02, 95% CI 0.65 to 1.39, *P* < 0.00001). Two trials (9.5%) reported BAA-related adverse events, and the most common adverse event was local subcutaneous ecchymosis.

**Conclusions:** BAA as an adjunctive therapy may have clinical benefits for improving allover motor function, upper limb motor function, lower limb motor function, and ADL in patients with PSMD. BAA-related adverse events were rare, tolerable, and recoverable. However, our review findings should be interpreted with caution because of the methodological weaknesses in the included trials. High-quality trials are needed to assess the adjunctive role of BAA in patients with PSMD.

## Introduction

Stroke is one of the most common causes of disability (1). The number of patients with stroke annually is increasing, as is the number of stroke-related deaths and disability-adjusted life-years lost (2). At least 5 million stroke survivors are left permanently disabled each year (3). The burden will be heavier in the next two decades due to an aging population and changing lifestyles (4). Among stroke survivors, motor dysfunction is the most common stroke sequelae. Hemiparesis patients account for approximately 85% of all stroke survivors, and approximately 55%-75% of stroke patients have persistent motor dysfunction (5). Poststroke motor dysfunction (PSMD) limits the patient's ability to move and negatively affects activities of daily living (ADL) (6). Therefore, more attention should be devoted to effectively improving the rehabilitation effect of PSMD.

Rehabilitation training (RT) plays an important role in the comprehensive rehabilitation treatment of stroke and can effectively promote the recovery of PSMD. RT can induce activity-dependent plasticity and promote recovery of motor function in stroke patients (7, 8). For example, Bobath therapy and exercise training, as common RT methods, can improve lower limb activities and balance capacities after stroke (9, 10). However, the effects of RT for PSMD are not very satisfactory (11). Although patients were given RT interventions, some patients within one year after stroke are still very likely to encounter bottlenecks, suffer from slow recovery, or even stagnation for a long time (12). In addition, RT is often performed one-to-one to achieve better therapeutic efficacy, but this will consume considerable labor and require a high degree of cooperation of patients for a long time. Due to the gap in medical conditions in different regions, the level of rehabilitation therapists is also significantly different, which affects the effect of RT. With RT alone for PSMD, the treatment duration is generally longer, and patient compliance is often poor. In recent years, increasing attention has been devoted to the application of alternative therapies in stroke rehabilitation.

Bo's abdominal acupuncture (BAA), in which acupuncturists choose abdominal acupoints to treat different diseases, is a new therapy in alternative and complementary medicine. Compared with RT or traditional acupuncture, there are several advantages of BAA in clinical practice. At first, patients will feel little or no pain during the treatment of BAA (13). Traditional acupuncture stresses the importance of needle sensations (namely, De Qi), while BAA does not consider the feelings of soreness, numbness, heaviness, or distention induced by the needle. As a result, patients easily accept treatment with BAA, and this approach has good compliance. Furthermore, the formulation and operation of BAAs are standardized (14, 15). The ruler is usually used to determine the location of abdominal acupoints (14, 15). We believe that these will not only ensure the accuracy of abdominal acupoint location but also the consistency of the effect by different practitioners in diverse regions. The higher repeatability and more practical popularity of BAA cannot be ignored. In China, BAA is often used to treat poststroke dysfunction within half a year, such as PSMD, shoulder-hand syndrome, fatigue, and so on.

However, until now, there has been no systematic review assessing the effect of BAA as an adjunctive therapy for patients with PSMD. Therefore, we conducted this review to assess whether BAA is an efficacious treatment modality for allover motor function using the Fugl-Meyer Assessment scale (FMA), upper limb motor function using the upper limb subscale of FMA (FMA-U), lower limb motor function using the lower limb subscale of FMA (FMA-L), and ADL using the Barthel Index (BI) in patients with PSMD. This study was registered on PROSPERO (No. CRD42017068718).

## Materials and Methods

### Types of Studies

We included all randomized controlled trials (RCTs) comparing BAA plus another therapy with the same other therapy alone (e.g., RT alone, conventional drugs (CD), body acupuncture (BA), and so on) in patients with PSMD. The publication status and language were not taken into account in the selection process of RCTs. We excluded crossover trials and RCTs that compared BAA with sham BAA or placebo in PSMD patients. We also excluded RCTs comparing different formulations and manufacturers of BAA.

### Participant Characteristics

Participants within six months after the onset of stroke were considered. Participants were over 18 years old, regardless of gender or race. Moreover, stroke must be diagnosed by internationally recognized criteria or diagnostic criteria adopted in China. Brain computerized tomography scanning (CT) or brain magnetic resonance imaging (MRI) as assisted diagnosis technology for stroke was necessary. Participants with subdural haematoma or subarachnoid hemorrhage were excluded.

### Types of Interventions

We included RCTs evaluating the efficacy and safety of BAA combined with another treatment in patients with PSMD. The duration and times of BAA were not taken into account. The treatment of the BAA group was BAA plus another therapy, while the treatment of the non-BAA group was the same other therapy alone. The other therapies mainly included RT, CD, BA, conventional acupuncture (CA), auricular acupuncture, scalp acupuncture, dry needle, fire needle, and Chinese herb medicine.

### Types of Outcome Measures

#### Primary Outcomes

The primary outcome was motor function and ADL. Allover motor function was assessed by the FMA, and upper and lower limb motor function was evaluated using FMA-U and FMA-L (16). The FMA scale contains 50 domains, and each domain was defined as 0 to 2 points. The highest score of the FMA is 100 points, including 66 points for the FMA-U and 44 points for the FMA-L. The higher the scores, the better the motor function (17). ADL were evaluated by BI or modified Barthel Index (MBI) (18). The MBI or BI contains 10 domains (e.g., eating, personal hygiene, bathing, toileting, dressing, anal control, bladder control, bed and chair transfer, level walking, and stairs). The total score of MBI is 0 to 100 points. The higher the scores, the ADL is better (19). The assessment time of the outcomes was at the end of the intervention.

#### Secondary Outcomes

The secondary outcome was mainly adverse events (AEs) related to BAA or other therapies. BAA-related AEs may include bleeding, infection, dizziness, local subcutaneous ecchymosis, and so on.

### Data Sources and Searches

We searched the following seven electronic databases from inception up to December 2020: Embase, PubMed, Cochrane Library, Chinese Biological Medicine Database (CBM), WAN FANG, Chinese Scientific Journal Database (VIP), and China National Knowledge Infrastructure (CNKI). The topic search was combined with the Cochrane highly sensitive search strategy for identifying RCTs. The search strategy was adjusted to search other databases (see Appendix 1). To find more eligible RCTs, we manually screened the reference lists of the included trials and all relevant reviews.

### Data Collection and Analysis

#### Selection of Studies

**Figure 1:**
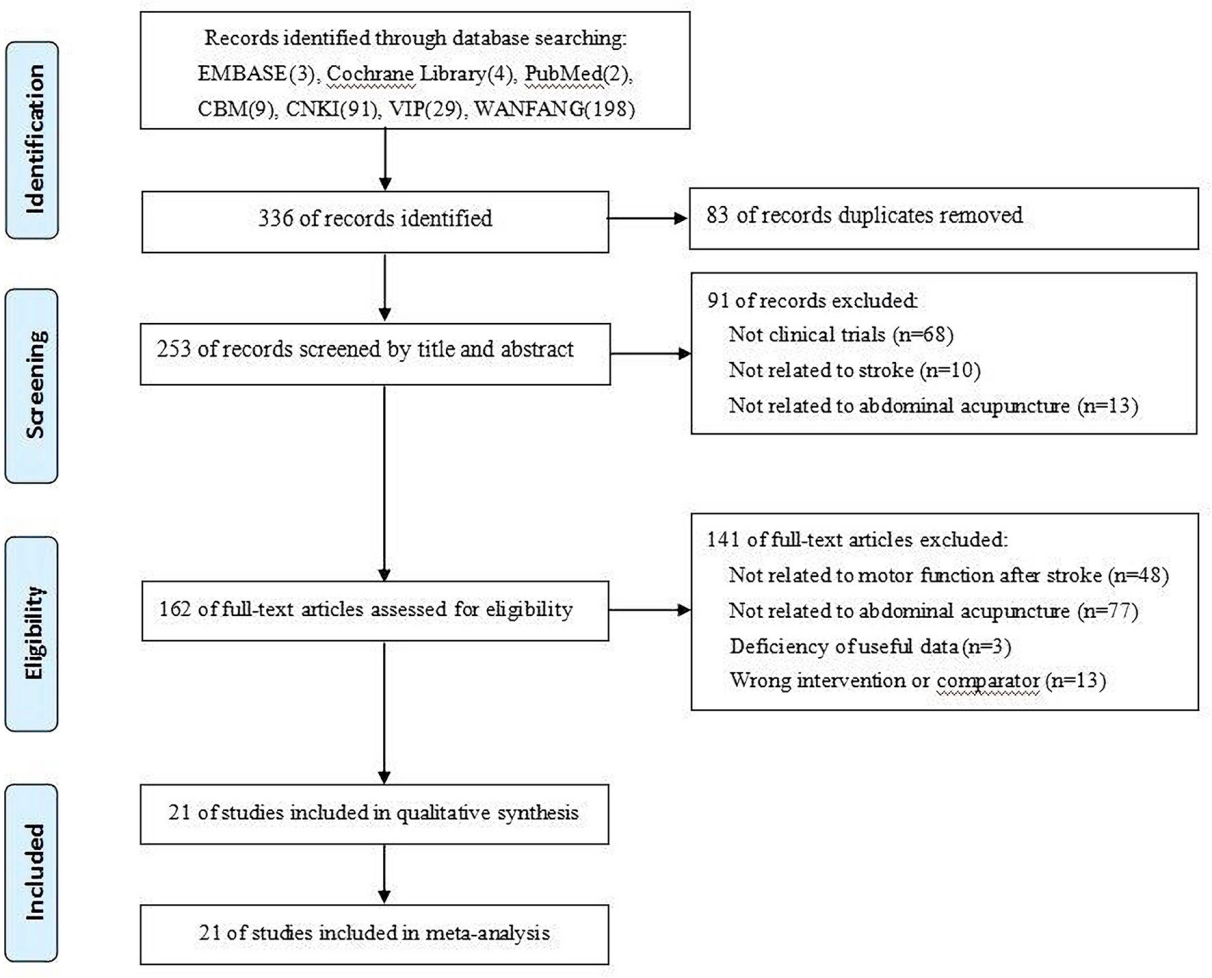
Study flow diagram.

The titles and abstracts of all articles obtained were reviewed by two independent authors based on the inclusion criteria. The full text of the potential articles was retrieved. Then, these two authors independently read the full articles to identify studies. The exclusion reasons of studies were recorded. Only the original published report of a study was retained. Moreover, disagreements about whether a study should be selected were discussed by two authors; if necessary, another author was consulted to reach a consensus. The selection flow of trials is shown in Figure 1.

#### Data Extraction and Management

Two authors independently used an Excel formatted table to extract data from the included studies. Then, they checked the accuracy of the information. The extracted information included author name, publication year, sample size, participants (e.g., gender, age), interventions, needle retention duration, the selected acupoints of BAA, outcomes, and so on. We contacted the study authors by e-mail to obtain missing information.

#### Quality Assessment

The methodological quality of each included study was assessed by two independent authors with the risk of bias (ROB) assessment tool in the *Cochrane Handbook for Systematic Reviews of Interventions* (20). The following domains were evaluated: sequence generation, allocation concealment, blinding, incomplete outcome data, selective outcome reporting, and other sources of bias. The ROB for each domain was graded as follows: low ROB, high ROB, or unclear. Disagreements on quality assessment were solved by consulting with another author. If more than half of the domains in a study had low ROB, the overall quality of the study was low risk.

#### Measures of Treatment Effect

For dichotomous outcomes, we used a risk ratio (RR) with 95% confidence intervals (CIs). For continuous data, we expressed the results as weighted mean differences (WMDs) with 95% CIs when outcomes were assessed by the same scale. If outcomes were measured by different scales, the standardized mean difference (SMD) with 95% CI was used instead of WMD.

#### Unit of Analysis Issues

We managed data for non-standard design studies under the guidelines recommended in the Cochrane Handbook for Systematic Reviews of Interventions 5.1.0.

#### Dealing With Missing Data

We contacted the study authors via e-mail to obtain missing data. When missing data were not obtained, we conducted statistical analyses using studies in which outcomes were reported.

#### Assessment of Heterogeneity

We compared the characteristics of patients (e.g., age, sex) and trial design (e.g., adequate sequence generation, blinding of assessors) to assess the clinical heterogeneity of the included studies. We also used the I-squared and Chi^2^ tests to evaluate the statistical heterogeneity of the included studies. We used a fixed effects model to pool the data of primary outcomes when I-squared <50%. If the heterogeneity was substantial (I-squared ≥ 50%), we used a random effects model to pool the data and attempted to interpret the results by subgroup analyses.

#### Assessment of Reporting Biases

If the number of included trials was more than ten, we used visually funnel plots and Egger's test to assess publication bias.

#### Data Synthesis

We performed statistical analyses using RevMan Version 5.3 (The Nordic Cochrane Center, the Cochrane Collaboration 2014) and Stata SE 14.0 (StataCorp LP, College Station, TX, USA). We conducted a meta-analysis for primary outcomes (e.g., FMA, FMA-U, FMA-L, and MBI) using a random effects model because of substantial heterogeneity. We were unable to obtain dichotomous data in this review. We performed a narrative summary for the secondary outcome (e.g., AEs).

#### Subgroup Analysis

We considered that the different add-on treatments to BAA may influence the adjunctive effect of BAA. We conducted subgroup analysis for primary outcomes as follows: BAA plus RT vs. RT alone and BAA plus BA vs. BA alone.

#### Sensitivity Analysis

We used random effects and fixed effects models to test the robustness of the meta-analysis. We also tested the robustness of the pooled results using specific methodological variables that could affect primary outcome measures (e.g., adequate sequence generation and blinding of assessors).

## Results

### Characteristics of Included Trials

Twenty-one trials (21–41) with 1,473 patients (740 patients in the BAA group) were included in this meta-analysis (Figure 1). Additional trials were not found by manually screening the reference lists of the included trials. All included trials were described as RCTs and published in Chinese from 2007 to 2020. The sample size ranged from 40 to 120; however, the calculation method of the sample was not described in any trials. The ratio of males and females between the included trials was similar, as was the average age. The treatment duration changed from 5 times to 30 times. Twelve trials (57.1%) (23–25, 29–33, 35, 36, 39, 40) compared BAA plus RT with RT alone, seven trials (33.3%) (22, 26–28, 34, 37, 41) compared BAA plus BA with BA alone, and one trial (4.8%) (21) compared BAA plus CA with CA alone. One trial (4.8%) (38) administered BAA and Xingnao kaiqiao acupuncture (XNKQ) to the BAA group, while the control group only received XNKQ. The general information of the included trials is shown in Table 1.

**Table 1:** General information of included trials.

**Study**	**Year**	**Sample size**	**Age (mean±SD)**	**Sex (male/female)**	**Regimen**	**Treatment Duration**	**Needle retention duration**	**The selected acupoints**	**Main outcomes**
Ding HM	2014	T:30	T:65 ± 6	T:14/16	T:BAA+CA	20 times, 4w	30 min	RN04, RN06, RN10, RN12, ST24, ST26, AB1, AB2, KI17	FMA-U
		C:30	C:66 ± 5	C:17/13	C:CA				
Guo SJ	2015	T:30	T:57.43 ± 11.15	T:22/8	T:BAA+BA	24 times, 4w	30 min	RN04, RN06, RN10, RN12, ST24, ST25, ST26, AB1, AB4, SP15	FMA, MBI
		C:30	C:53.67 ± 9.67	C:23/7	C:BA				
Hao SF	2015	T:30	NR	41/19	T:BAA+RT	10 times, 2w	30 min	RN04, RN06, RN10, RN12, ST24, ST26, AB4, SP15	FMA, MBI
		C:29			C:RT				
Jin LQ	2018	T:60	T:56.43 ± 9.84	T:34/26	T:BAA+RT	24 times, 8w	30 min	RN04, RN06, RN10, RN12, ST24, ST26, AB1, AB2, AB4, AB6, AB7, KI17	FMA
		C:60	C:58.60 ± 7.33	C:36/24	C:RT				
Kong L	2019	T:47	T:58.62 ± 8.41	T:28/19	T:BAA+RT	30 times, 30d	20 min	RN04, RN06, RN10, RN12, ST24, ST25, ST26, AB1, AB2, AB4, AB6, SP15	FMA, BI
		C:47	C:65.02 ± 8.34	C:29/18	C:RT				
Li WJ	2012	T:20	T:65.50 ± 2.39	T:13/7	T:BAA+BA	20 times, 4w	30 min	RN04, RN06, RN10, RN12, ST24, ST26, AB1, AB4	FMA-L, MBI
		C:20	C:65.85 ± 2.85	C:15/5	C:BA				
Li JY	2012	T:31	T:63.23 ± 9.98	T:NR	T:BAA+BA	30 times, 6w	30 min	RN04, RN06, RN10, RN12, ST24, ST26, AB1, AB4, KI13, SP15	MBI
		C:31	C:66.58 ± 12.34	C:NR	C:BA				
Li YQ	2014	T:45	T:63.64 ± 9.33	T:26/19	T:BAA+BA	30 times, 6w	30 min	RN04, RN06, RN10, RN12, ST24, ST26, AB1, AB4, SP15, KI13	FMA-U, FMA-L
		C:45	C:64.66 ± 8.52	C:24/21	C:BA				
Ling SS	2019	T:22	T:63.18 ± 9.93	T:17/5	T:BAA+RT	20 times, 4w	30 min	RN04, RN06, RN10, RN12, ST24, AB1, AB2, KI17	FMA-U, MBI
		C:22	C:63.09 ± 9.65	C:13/9	C:RT				
Qiu LF	2018	T:30	T:57.90 ± 8.75	T:18/12	T:BAA+RT	24 times, 4w	30 min	RN04, RN06, RN10, RN12, ST24, ST25, ST26, AB1, AB2, AB4, KI17	FMA-U, MBI
		C:30	C:59.80 ± 9.68	C:17/13	C:RT				
Su CX	2017	T:20	T:58.4 ± 4.8	T:14/6	T:BAA+RT	20 times, 4w	30 min	RN04, RN06, RN10, RN12, ST24, ST26, AB1, AB4	FMA
		C:20	C:56.7 ± 5.2	C:13/7	C:RT				
Wang CX	2015	T:40	T:66.3 ± 6.1	T:21/19	T:BAA+RT	30 times, 30d	20 min	RN04, RN06, RN10, RN12, ST24, ST25, ST26, AB1, AB2, AB4, AB6, SP15	FMA, BI
		C:40	C:65.5 ± 7.6	C:20/20	C:RT				
Wang JH	2020	T:45	T:65.48 ± 3.32	T:29/16	T:BAA+RT	5 times	30 min	RN04, RN06, RN10, RN12, ST24, ST26, AB1, AB2, AB4, AB6, AB7, KI17	MBI
		C:45	C:65.36 ± 3.21	C:28/17	C:RT				
Wang LP	2007	T:38	T:51–78	T:30/8	T:BAA+BA	15 times	30 min	RN04, RN06, RN10, RN12, ST24, ST26, AB1, AB2, AB4, AB6, AB7, KI17	FMA, MBI
		C:38	C:50–75	C:27/11	C:BA				
Wang QW	2014	T:47	T:63.2 ± 7.9	T:24/23	T:BAA+RT	40 times, 8w	20 min	RN04, RN06, RN10, RN12, ST24, ST26, AB1, AB4	FMA, MBI
		C:43	C:65.2 ± 8.9	C:22/21	C:RT				
Wang YH	2016	T:30	T:66 ± 6	T:16/14	T:BAA+RT	30 times, 30d	20 min	RN04, RN06, RN10, RN12, ST24, ST25, ST26, AB1, AB2, AB4, AB6, SP15	FMA-U, FMA-L
		C:30	C:66 ± 8	C:14/16	C:RT				
Wang YJ	2008	T:49	T:64.73 ± 9.81	T:28/21	T:BAA+BA	15 times, 3w	30 min	RN04, RN06, RN10, RN12, ST24, ST26, AB1, AB4, SP15	FMA, FMA-U, FMA-L, AE
		C:48	C:62.88 ± 10.17	C:29/19	C:BA				
Wu N	2013	T:36	T:55.80 ± 9.92	T:22/14	T:BAA+XNKQ	20 times, 4w	30 min	RN04, RN06, RN10, RN12, ST24, ST26, AB1, AB4, SP15	FMA, MBI
		C:36	C:54.92 ± 10.56	C:20/16	C:XNKQ				
Wu N	2014	T:30	T2:56.46 ± 7.37	T:18/12	T:BAA+RT	20 times, 4w	30 min	RN04, RN06, RN10, RN12, ST24, ST25, ST26, AB4, SP15	FMA, MBI
		C:29	C:57.08 ± 6.31	C:19/10	C:RT				
Xie RM	2011	T:30	66.58 ± 9. 53	40/20	T:BAA+RT	20 times, 4w	30 min	RN04, RN06, RN10, RN12, ST24, ST26, AB1, AB2, AB4, AB6, SP15	FMA, BI
		C:30			C:RT				
Xu ZQ	2011	T:30	T:63.88 ± 9.53	T:16/14	T:BAA+BA	15 times	30 min	RN04, RN06, RN10, RN12, ST24, ST26, AB1, AB4, SP15	FMA, MBI, AE
		C:30	C:64.55 ± 10.61	C:17/13	C:BA				

*AE, adverse event; BI, Barthel Index; BA, body acupuncture; BAA, Bo's abdominal acupuncture; C, control group; CA, conventional acupuncture; d, day; FMA, Fugl-Meyer Assessment Scale; FMA-U, Fugl-Meyer Assessment Scale for upper extremity; FMA-L, Fugl-Meyer Assessment Scale for lower extremity; MBI, modified Barthel Index; min, minutes; NR, not referred; RT, rehabilitation training; T, treatment group; w, week; XNKQ, Xingnao kaiqiao acupuncture*.

### Risk of Bias Within Trials

**Figure 2:**
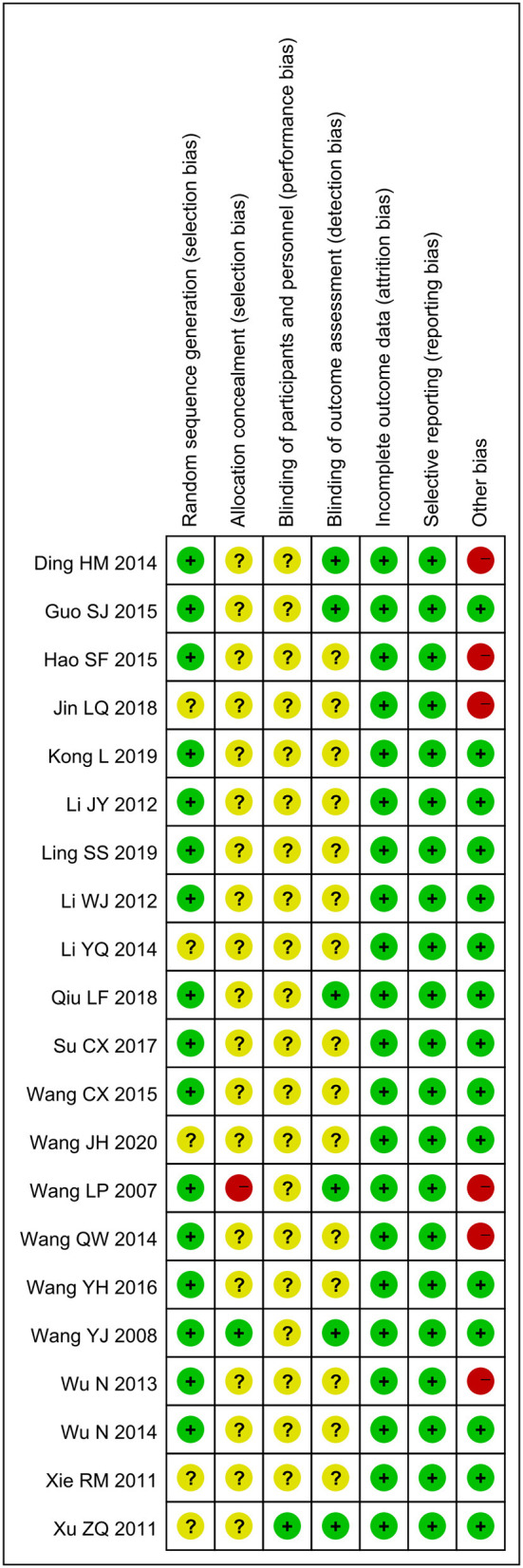
Risk of bias assessments of included studies.

Sixteen trials (76.2%) (21–23, 25–27, 29–32, 34–39) with a low ROB in random sequence generation reported that the random sequences were generated by random number tables or computer software. However, five trials (23.8%) (24, 28, 33, 40, 41) did not make a detailed report about the method of random sequence generation. Only one trial (4.8%) (37) reported allocation concealment. Most of the trials did not describe methods of blinding, but six trials (28.6%) (21, 22, 30, 34, 37, 41) reported blinding of outcome assessment, and one trial (4.8%) (41) reported investigators were blind for group allocation. Three trials (14.3%) (37, 39, 41) reported drop-outs, but none of them conducted intention-to-treat analyses. In terms of selective reporting outcome, it was awfully difficult to judge because the protocols of included trials were not found. According to the descriptions in the methods section of the included trials, all trials were assessed to be at low ROB. In other sources of bias, six trials (28.6%) (21, 23, 24, 34, 35, 38) had a high ROB due to inadequate or incorrect statistical methods. In other words, the overall quality of the 14 trials (66.7%) included was low risk (Figure 2).

### Meta-Analysis

#### Fugl-Meyer Assessment Scale (FMA)

**Figure 3:**
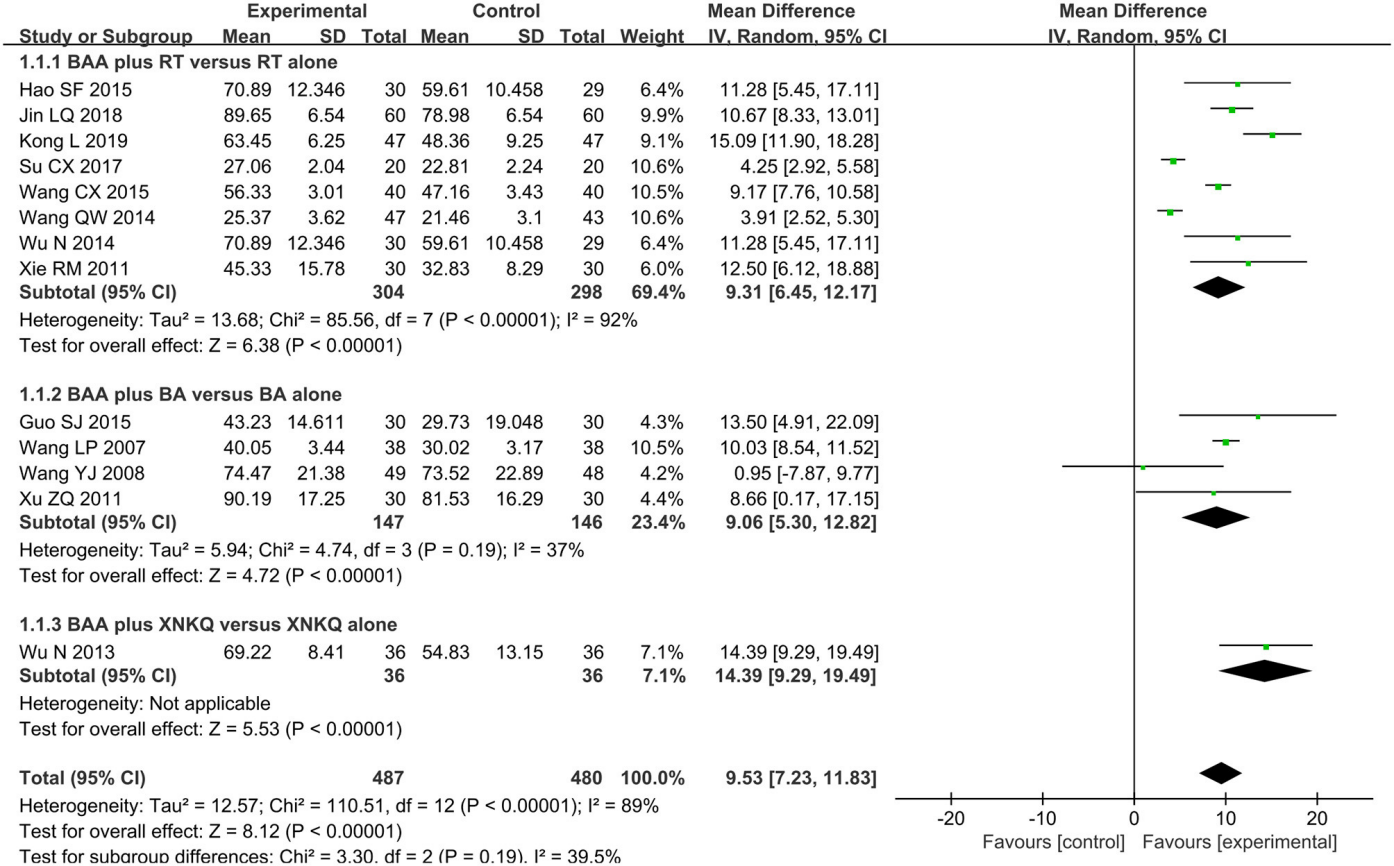
Forest plot and meta-analysis of FMA. (BA, body acupuncture; BAA, Bo's abdominal acupuncture; CI, confidence interval; FMA, Fugl-Meyer Assessment Scale; RT, rehabilitation training; XNKQ, Xingnao kaiqiao acupuncture).

FMA as the primary outcome was reported in thirteen trials (61.9%) (22–25, 31, 32, 34, 35, 37–41) with 967 participants. Because of significant heterogeneity, the effect of BAA on FMA between the BAA and non-BAA groups was analyzed by a random effects model. The improvement in FMA in the BAA group was better than that in the non-BAA group (WMD 9.53, 95% CI 7.23 to 11.83, *P* < 0.00001) (Figure 3).

#### FMA for Upper Extremity (FMA-U)

**Figure 4:**
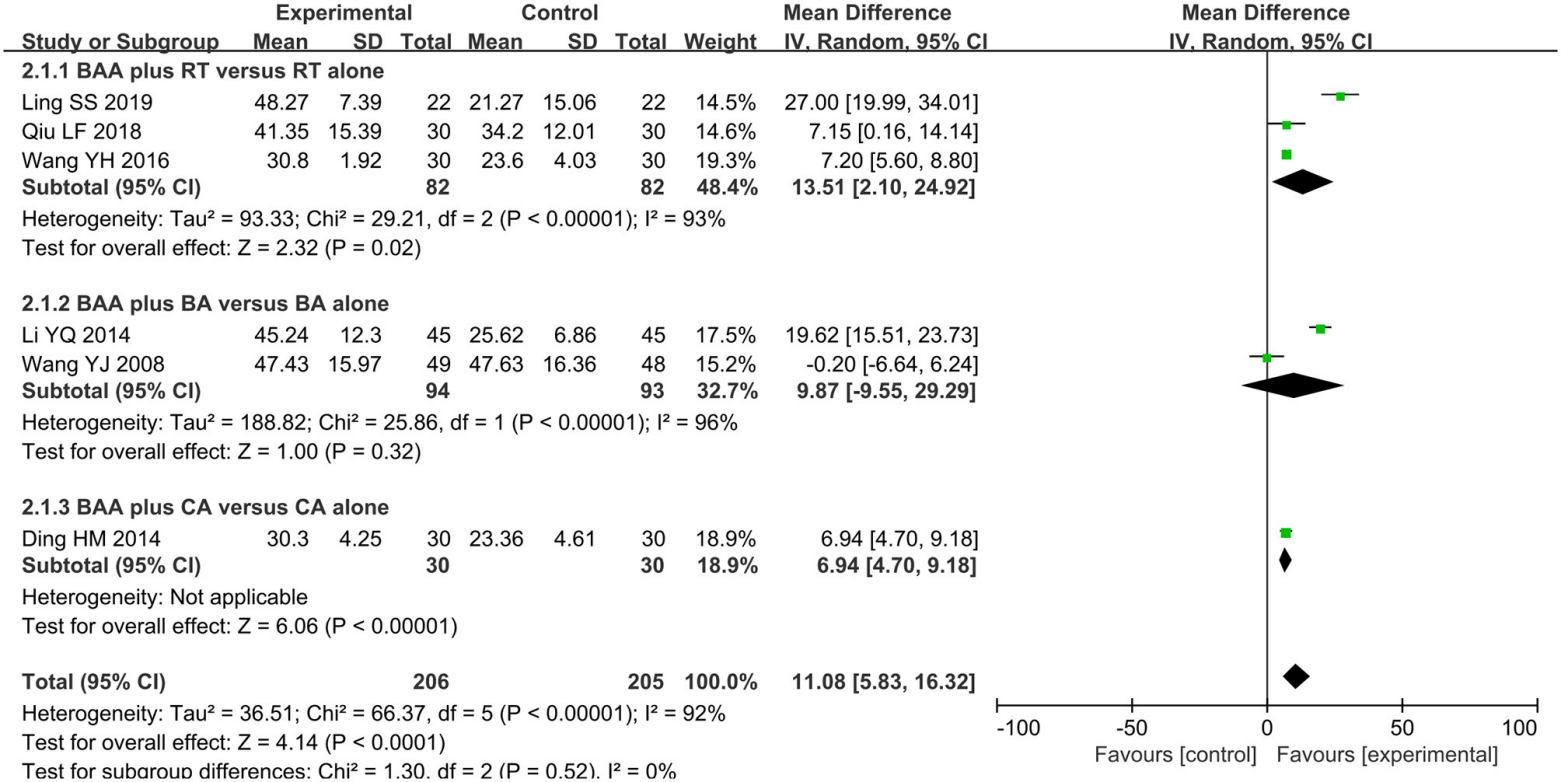
Forest plot and meta-analysis of FMA-U. (BA, body acupuncture; BAA, Bo's abdominal acupuncture; CA, conventional acupuncture; CI, confidence interval; FMA-U, Fugel-Meyer Assessment Scale for upper extremity; RT, rehabilitation training).

The motor function of the upper extremity in six trials (28.6%) (21, 28–30, 36, 37) with 411 patients was evaluated using FMA-U. The effect on FMA-U was analyzed by a random effects model due to significant heterogeneity, and a significant difference in FMA-U was found between the EA and non-EA groups (WMD 11.08, 95% CI 5.83 to 16.32, *P* < 0.0001) (Figure 4).

#### FMA for Lower Extremity (FMA-L)

**Figure 5:**
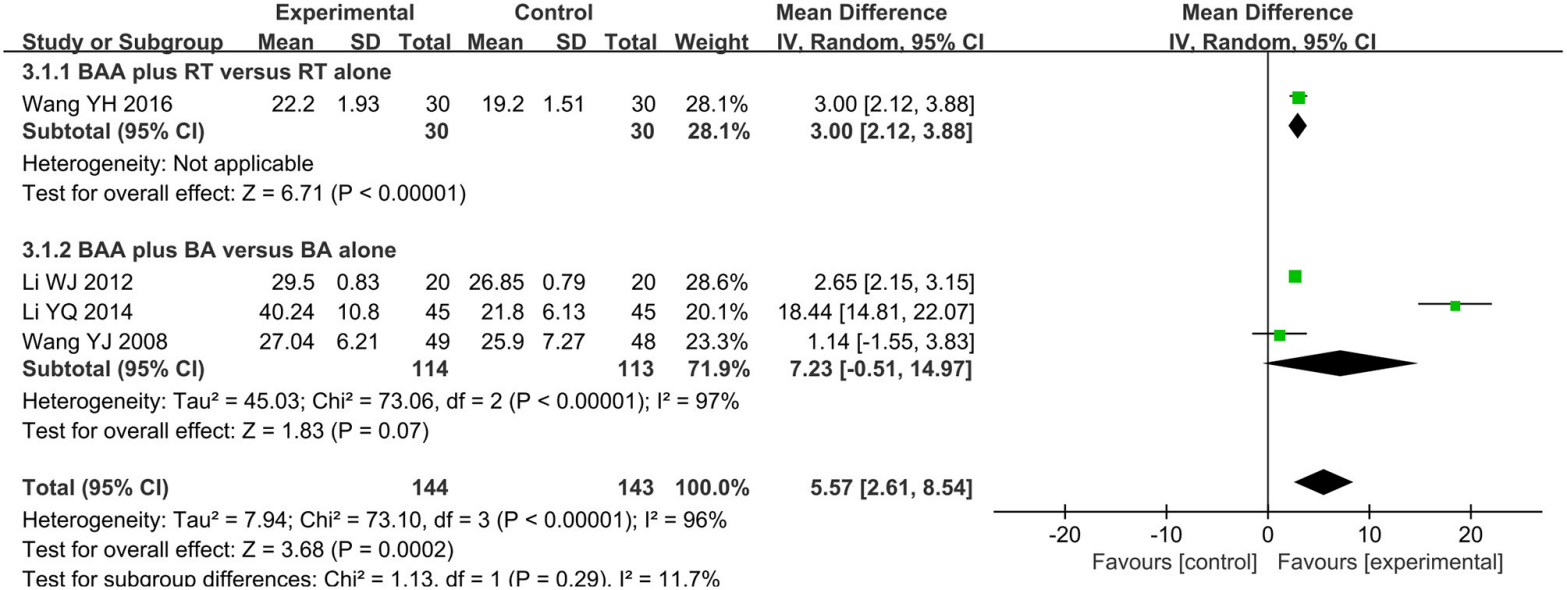
Forest plot and meta-analysis of FMA-L. (BA, body acupuncture; BAA, Bo's abdominal acupuncture; CI, confidence interval; FMA-L, Fugel-Meyer Assessment Scale for lower extremity; RT, rehabilitation training).

Four trials (19.1%) (26, 28, 36, 37) with 287 participants used FMA-L to assess lower limb motor function. The effect on FMA-L was analyzed by a random effects model, and the difference between the BAA group and non-BAA group was obvious (WMD 5.57, 95% CI 2.61 to 8.54, *P* = 0.0002) (Figure 5).

#### Activities of Daily Living (ADL)

**Figure 6:**
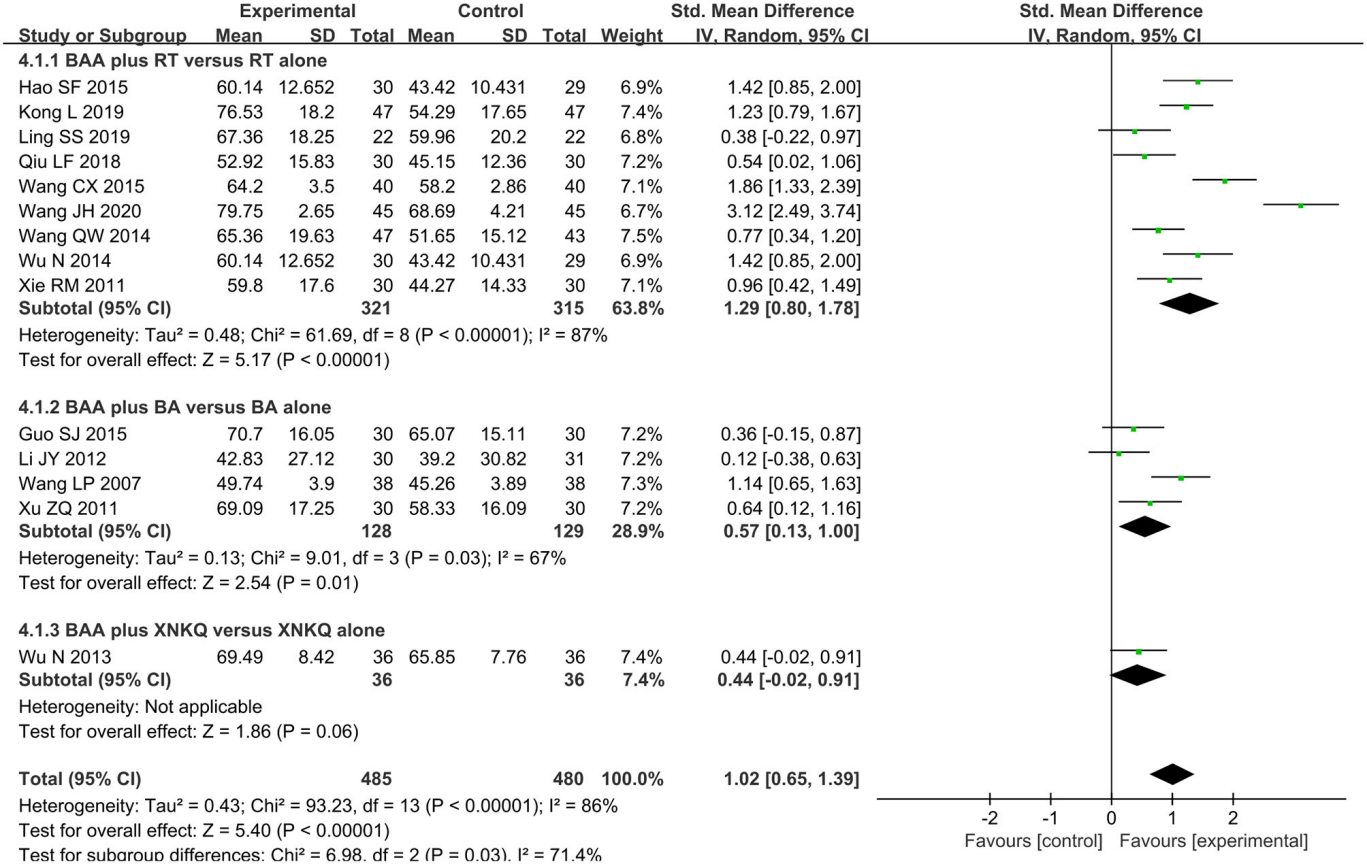
Forest plot and meta-analysis of MBI. (BA, body acupuncture; BAA, Bo's abdominal acupuncture; CI, confidence interval; MBI, modified Barthel Index; RT, rehabilitation training; XNKQ, Xingnao kaiqiao acupuncture).

ADL was assessed by MBI or BI in fourteen trials (66.7%) (22, 23, 25, 27, 29, 30, 32–35, 38–41) with 965 participants. The effect of BAA on ADL between the BAA and non-BAA groups was evaluated by a random effects model, owing to significant heterogeneity. In terms of the improvement of ADL, there was a significant difference between the two groups (SMD 1.02, 95% CI 0.65 to 1.39, *P* < 0.00001) (Figure 6).

### Adverse Events

Two trials (9.5%) (37, 41) reported treatment-related adverse events. One trial (41) reported three cases in the BAA group experiencing local subcutaneous ecchymosis, and the other trial (37) reported five cases in the BAA group and eight cases in the non-BAA group suffering from local subcutaneous ecchymosis. Fortunately, the symptoms were relieved after putting warm wet towels on local skin for one to seven days.

### Subgroup Analysis

#### BAA Plus RT vs. RT Alone

Eight trials (38.1%) (23–25, 31, 32, 35, 39, 40) with 602 participants used FMA as an outcome measure of allover motor function. Three trials (14.3%) (29, 30, 36) used FMA-U to assess the upper limb motor function of 164 patients, and one trial (4.8%) (36) with 60 patients used FMA-L to evaluate lower limb motor function. The effects on FMA, FMA-U, and FMA-L were analyzed by a random effects model due to significant heterogeneity. There was a significant difference in FMA between BAA plus RT and RT alone (WMD 9.31, 95% CI 6.45 to 12.17, *P* < 0.00001). BAA plus RT for the improvement of FMA-U and FMA-L was better than RT alone (WMD 13.51, 95% CI 2.10 to 24.92, *P* = 0.02 and WMD 3.00, 95% CI 2.12 to 3.88, *P* < 0.00001, respectively) (Figures 3–5).

Nine trials (42.9%) (23, 25, 29, 30, 32, 33, 35, 39, 40) with 636 participants applied MBI or BI to assess the change in ADLs. Due to statistical heterogeneity, meta-analyses with a random effects model were conducted to assess the effect on MBI in this subgroup analysis. In the comparison of BAA plus RT and RT alone, a difference in MBI existed (SMD 1.29, 95% CI 0.80 to 1.78, *P* < 0.00001) (Figure 6).

#### BAA Plus BA vs. BA Alone

Four trials (19.1%) (22, 34, 37, 41) with 293 patients used FMA to compare the effectiveness of BAA plus BA against BA alone. Meta-analyses with a random effects model showed that there was a significant difference in these trials (WMD 9.06, 95% CI 5.30 to 12.82, *P* < 0.00001) (Figure 3). Two trials (9.5%) (28, 37) used FMA-U to evaluate the upper limb motor function of 187 patients. Three trials (14.3%) (26, 28, 37) applied FMA-L to assess the lower limb motor function of 227 patients. A random effects model was utilized to analyse the effect on FMA-U and FMA-L because of significant heterogeneity. There was no significant difference in FMA-U and FMA-L between BAA plus BA and BA alone (WMD 9.87, 95% CI −9.55 to 29.29, *P* = 0.32 and WMD 7.23, 95% CI −0.51 to 14.97, *P* = 0.07, respectively) (Figures 4, 5).

Four trials (19.1%) (22, 27, 34, 41) used MBI to assess the ADL of 257 participants with PSMD. Meta-analyses with a random effects model showed that BAA plus BA for the improvement of ADLs was better than BA alone (SMD 0.57, 95% CI 0.13 to 1.00, *P* = 0.01) (Figure 6).

### Sensitivity Analysis

Random effects and fixed effects models were used to test the robustness of the meta-analysis because different statistical models may influence the results. There was no significant difference between the different statistical models (Table 2). Moreover, the robustness of the meta-analysis was also tested by using specific methodological variables that could affect the outcome measures (adequate sequence generation and blinding of assessors) (Table 2).

**Table 2:** Results of sensitivity analysis.

**Study type**	**Studies, no**.	**Participants, no**		**Study heterogeneity**				**Analysis model**	**MD (95% CI)**	***p* value**
		**Experiment group**	**Control group**	**Chi^**2**^**	**df**	**I^**2**^, %**	***p* value**			
*Fugl-Meyer Assessment scale (FMA)*										
BAA vs. non BAA	13	487	480	110.51	12	89	<0.00001	random	9.53 (7.23, 11.83)	<0.00001
								fixed^*^	7.59 (6.95, 8.22)	<0.00001
BAA plus RT vs. RT alone	8	304	298	85.56	7	92	<0.00001	random	9.31 (6.45, 12.17)	<0.00001
								fixed^*^	6.88 (6.17, 7.60)	<0.00001
BAA plus BA vs. BA alone	4	147	146	4.74	3	37	0.19	random	9.06 (5.30, 12.82)	<0.00001
								fixed^*^	9.85 (8.42, 11.27)	<0.00001
*FMA for upper extremity (FMA-U)*										
BAA vs. non BAA	6	206	205	66.37	5	92	<0.00001	random	11.08 (5.83, 16.32)	<0.0001
								fixed^*^	8.47 (7.28, 9.65)	<0.00001
BAA plus RT vs. RT alone	3	82	82	29.21	2	93	<0.00001	random	13.51 (2.10, 24.92)	0.02
								fixed^*^	8.13 (6.61, 9.65)	<0.00001
BAA plus BA vs. BA alone	2	94	93	25.86	1	96	<0.00001	random	9.87 (-9.55, 29.29)	0.32
								fixed^*^	13.87 (10.40, 17.34)	<0.00001
*FMA for lower extremity (FMA-L)*										
BAA vs. non BAA	4	144	143	73.10	3	96	<0.00001	random	5.57 (2.61, 8.54)	0.0002
								fixed^*^	2.91 (2.49, 3.34)	<0.00001
*ACTIVITIES of daily living (ADL)*										
BAA vs. non BAA	14	485	480	93.23	13	86	<0.00001	random	1.02 (0.65, 1.39)^#^	<0.00001
								fixed^*^	0.97 (0.83, 1.10)^#^	<0.00001
BAA plus RT vs. RT alone	9	321	315	61.69	8	87	<0.00001	random	1.29 (0.80, 1.78)^#^	<0.00001
								fixed^*^	1.23 (1.05, 1.40)^#^	<0.00001
BAA plus BA vs. BA alone	4	128	129	9.01	3	67	0.03	random	0.57 (0.13, 1.00)^#^	0.01
								fixed^*^	0.57 (0.32, 0.83)^#^	<0.00001
*Trials with adequate sequence generation:*										
BAA vs. non BAA (FMA)	10	367	360	100.69	9	91	<0.00001	random^*^	9.23 (6.59, 11.87)	<0.00001
BAA vs. non BAA (FMA-U)	5	161	160	35.61	4	89	<0.00001	random^*^	9.11 (4.34, 13.87)	0.0002
BAA vs. non BAA (FMA-L)	3	99	98	1.78	2	0	0.41	fixed^*^	2.70 (2.27, 3.13)	<0.00001
BAA vs. non BAA (MBI)	11	380	375	44.15	10	77	<0.00001	random^*^	0.88 (0.56, 1.20)^#^	<0.00001
*Trials with adequate blinding of assessors:*										
BAA vs. non BAA (FMA)	4	147	146	4.74	3	37	0.19	fixed^*^	9.85 (8.42, 11.27)	<0.00001
BAA vs. non BAA (FMA-U)	3	109	108	4.29	2	53	0.12	fixed^*^	6.25 (4.22, 8.28)	<0.00001
BAA vs. non BAA (MBI)	4	128	128	5.26	3	43	0.15	fixed^*^	0.68 (0.43, 0.93)^#^	<0.00001

*BA, body acupuncture; BAA, Bo's abdominal acupuncture; CI, confidence interval; df, degrees of freedom; RT, rehabilitation training; WMD, weight mean difference*.

# *Presented as standardized mean difference (SMD);*

* *Represents the meta-analysis results was not shown in the figures*.

#### All Trials With Adequate Sequence Generation

Sixteen trials (76.2%) (21–23, 25–27, 29–32, 34–39) with 1,053 patients used adequate sequence generation. Ten of the sixteen trials (47.6%) (22, 23, 25, 31, 32, 34, 35, 37–39) used FMA to assess the recovery of overall motor function. A significant difference was found between the BAA and non-BAA groups (WMD 9.23, 95% CI 6.59 to 11.87, *P* < 0.00001) (Table 2). Five trials (23.8%) (21, 29, 30, 36, 37) with 321 patients used the FMA-U to assess upper limb motor function. A significant difference was also found between the BAA and non-BAA groups (WMD 9.11, 95% CI 4.34 to 13.87, *P* = 0.0002) (Table 2). Three trials (14.3%) (26, 36, 37) with 197 patients used the FMA-L to assess lower limb motor function. There was a significant difference between the BAA group and the non-BAA group (WMD 2.70, 95% CI 2.27 to 3.13, *P* < 0.00001) (Table 2). Eleven trials (52.4%) (22, 23, 25, 27, 29, 30, 32, 34, 35, 38, 39) with 755 patients used the MBI or BI to assess the improvement of ADL. A significant difference was found between the BAA and non-BAA groups (SMD 0.88, 95% CI 0.56 to 1.20, *P* < 0.00001) (Table 2).

#### All Trials With Blinded Assessors

Six trials (28.6%) (21, 22, 30, 34, 37, 41) with 413 patients used blinded assessors. Four of the six trials (19.1%) (22, 34, 37, 41) used FMA to assess the allover motor function of 293 patients with PSMD. Three trials (14.3%) (21, 30, 37) with 217 patients applied FMA-U to evaluate upper limb motor function. There was a significant difference in FMA and FMA-U between the BAA group and the non-BAA group (WMD 9.85, 95% CI 8.42 to 11.27, *P* < 0.00001 and WMD 6.25, 95% CI 4.22 to 8.28, *P* < 0.00001, respectively) (Table 2). One trial (4.8%) (37) with 97 patients used the FMA-L to assess lower limb motor function, and there was a significant difference between the two groups. Four trials (19.1%) (22, 30, 34, 41) with 256 participants applied MBI or BI to evaluate the change in ADLs. A fixed effects model was selected to analyse the effect on MBI. The result favored the BAA group (SMD 0.68, 95% CI 0.43 to 0.93, *P* < 0.00001) (Table 2).

### Publication Bias

**Figure 7:**
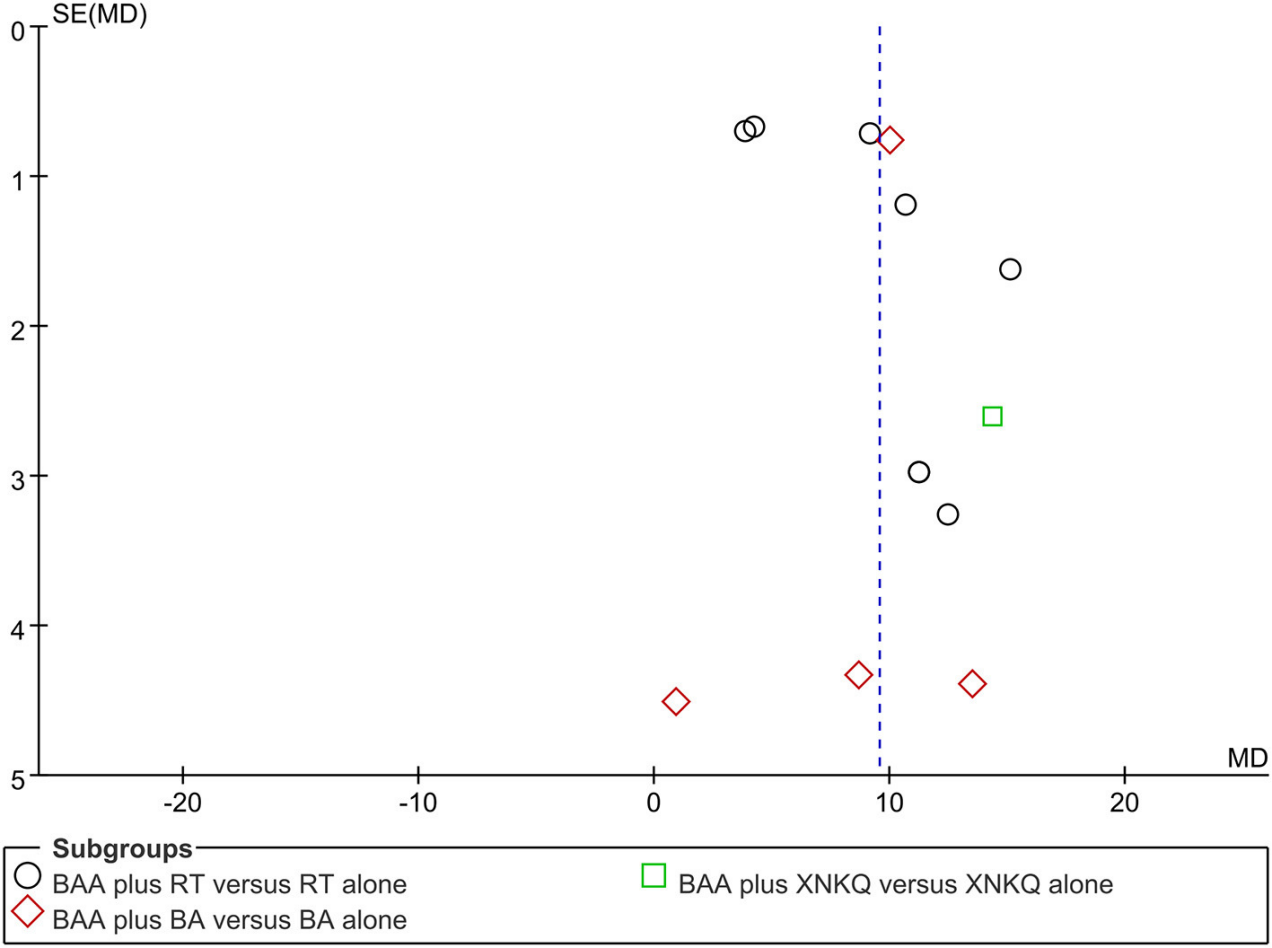
Funnel plots illustrating meta-analysis of FMA. (BA, body acupuncture; BAA, Bo's abdominal acupuncture; FMA, Fugl-Meyer Assessment Scale; WMD, weight mean difference; RT, rehabilitation training; SE, standard error; XNKQ, Xingnao kaiqiao acupuncture).

**Figure 8:**
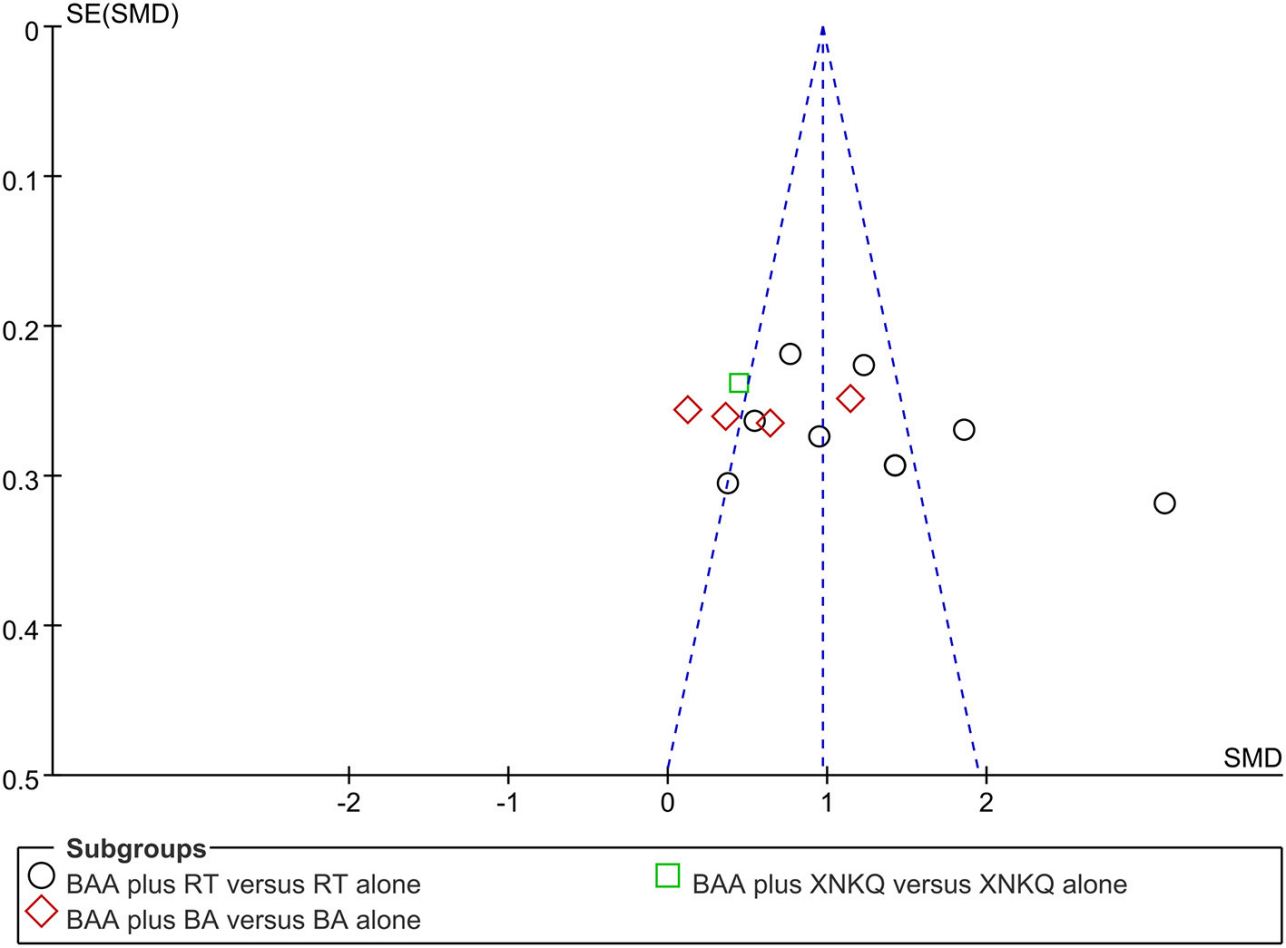
Funnel plots illustrating meta-analysis of MBI. (BA, body acupuncture; BAA, Bo's abdominal acupuncture; MBI, modified Barthel Index; SMD, standardized mean difference; SE, standard error; RT, rehabilitation training; XNKQ, Xingnao kaiqiao acupuncture).

Funnel plots and Egger's tests were used to assess publication bias based on FMA and MBI. Egger's tests showed that there were publication biases for the included trials of FMA (*P* = 0.039) but not MBI (*P* = 0.140). Some trials did not lie inside the 95% CI, and the distribution was located in imbalance. The results indicated potential publication bias (Figures 7, 8).

## Discussion

This meta-analysis of twenty-one RCTs with 1,473 patients compared the efficacy and safety of BAA plus another therapy with the same other therapy alone. Our findings from this review show that adjunctive BAA was beneficial for improving motor function and ADL. According to the results of the subgroup analysis, compared with RT alone or BA alone, BAA plus RT or BA had more advantages in improving overall motor function, lower limb motor function, and ADL. Two trials (9.5%) reported BAA-related AEs, and the main AE was local subcutaneous ecchymosis. The results of sensitivity analysis indicated that the effects of BAA plus other therapies on motor function and ADL of patients with PSMD were robust.

In terms of clinical implications, our findings indicated that BAA plus other therapies may benefit the recovery of motor function and ADL in PSMD patients. Furthermore, BAA-related AEs were rare, tolerable, and recoverable. However, due to the methodological weaknesses of the included trials, the findings from this meta-analysis should be considered with caution.

BAA as an alternative and complementary therapy has been used to treat different diseases for decades. Professor Bo established BAA according to Shenque Buqi theory, Zang-Fu theory, and meridian theory in the 1960s (42). Due to the characteristics of the multilevel spatial structure of the abdomen, the different needling depths of the BAA can have different effects (43). Furthermore, the therapeutic effect of abdominal acupoints is not limited to local problems but also to improve the whole body. In the theory of BAA, it can activate the body's congenital and acquired meridian systems and regulate the production and distribution of qi and blood (42). A previous study used functional magnetic resonance imaging (fMRI) to find that BAA can not only improve the cognitive network function of the central nervous system but also promote the functional reorganization and plasticity of the cerebral cortex (44). These are potential mechanisms of BAA in treating motor dysfunction and ADL impairment following stroke.

Numerous studies have reported that acupuncture combined with other therapies may benefit the treatment of poststroke dysfunction (45–47). However, Frank et al. (48) found that with stroke rehabilitation, acupuncture has no additional effect on motor recovery but has a small positive effect on disability in PSMD patients within six months. Our previous review showed that electroacupuncture combined with RT and/or conventional drugs may be a benefit for PSMD within 2 weeks (49). However, BAA as a style of acupuncture was not included in these reviews. The adjunct efficacy of BAA for PSMD is still unclear. This meta-analysis focused on the efficacy of BAA as an adjunctive therapy for the recovery of motor function and ADL in PSMD patients within six months after stroke. The results show that BAA as an adjunctive therapy may have clinical benefits for improving motor function and ADL in patients with PSMD.

To the best of our knowledge, this is the first systematic review to evaluate the efficacy and safety of BAA as an adjunctive therapy for improving motor function and ADL in PSMD patients within six months following stroke. We rigorously conducted this meta-analysis following Cochrane Collaboration guidelines. We extensively searched the eligible RCTs in seven electronic databases from inception up to December 2020 and manually screened the reference lists of the included trials and all relevant reviews. We also performed the selection process following PRISMA guidelines. Two authors independently conducted the study selection, data extraction, and quality assessment. Furthermore, the studies included in this meta-analysis were RCTs comparing BAA plus another therapy with the same other therapy alone. The FMA and MBI, as the primary outcome measures, have been widely used in the clinic for the assessment of limb motor function and ADL in stroke patients. These findings ensure a more accurate assessment of the adjunctive efficacy of BAA in patients with PSMD. Whether the standard stroke rehabilitation program for PSMD should be combined with or without BAA is a hot topic. To some extent, our findings provide evidence for the clinical application of BAA in PSMD patients.

However, this systematic review has several limitations that should be considered. First, all included trials were performed in China and published in Chinese, which may lead to selection bias and limit the generalization of the results. Although we tried our best to search the related literature, some published or unpublished trials may have not been identified. Few trials with negative results have been reported. The results of the funnel plots and Egger's tests on FMA and ADL showed a potential publication bias. Second, the sample size of the included trials was relatively small. The maximum sample number was 120 cases, and the minimum sample number was 40 cases. The treatment duration of all included trials was not consistent. The shortest duration was five times, and the longest duration was forty times. Third, five trials (23.8%) (24, 28, 33, 40, 41) were unclear or uncorrected about the method of random sequence generation. Ninety-five percent of the included trials did not report allocation concealment or blinding of the investigator. Only six trials (28.6%) (21, 22, 30, 34, 37, 41) reported blinding of outcome assessment. A total of 28.6% of the trials used inadequate or incorrect statistical methods. Three trials (14.3%) (37, 39, 41) reported dropout; however, none of them conducted intention-to-treat analyses. These factors may lead to the observed heterogeneity, which limits the reliability of the results. We suggest that large samples and rigorously designed studies with long-term follow-up should be conducted to confirm the adjunctive efficacy of BAA for PSMD in the future.

## Conclusion

BAA as an adjunctive therapy may have clinical benefits for improving allover motor function, upper limb motor function, lower limb motor function, and ADL in patients with PSMD. BAA-related adverse events were rare, tolerable, and recoverable. However, our review findings should be considered with caution because of the methodological weaknesses in the included trials. High-quality trials are needed to assess the adjunctive role of BAA for patients with PSMD.

## Data Availability Statement

The original contributions presented in the study are included in the article/Supplementary Material, further inquiries can be directed to the corresponding author/s.

## Author Contributions

JZ, LZ, and LL are responsible for conception and design of this systematic review. The manuscript of this article was drafted by JZ and revised by LZ and LL. The search strategies were designed by JZ and LL. The electronic search was conducted by BX, PZ, and YW. YT manually screened the reference lists of the included trials and all relevant reviews. JZ and BX extracted data. The risk of bias was assessed by JZ and PZ, independently. JZ and LL analyzed and interpreted the data. LZ and LL arbitrated any disagreements in the process of systematic review. All authors approved this manuscript.

## Funding

This study was funded by the special project of Lingnan modernization of traditional Chinese medicine in 2019 Guangdong Provincial R & D Program (No. 2020B1111100008), the Chinese Medicine Innovation Team Project of the State Administration of Traditional Chinese Medicine, and the project of Traditional Chinese Medicine Bureau of Guangdong Province (No. 20201153). The funders had no influence on study design, data collection and analysis, decision to publish, or preparation of the manuscript.

## Conflict of Interest

The authors declare that the research was conducted in the absence of any commercial or financial relationships that could be construed as a potential conflict of interest.

## Publisher's Note

All claims expressed in this article are solely those of the authors and do not necessarily represent those of their affiliated organizations, or those of the publisher, the editors and the reviewers. Any product that may be evaluated in this article, or claim that may be made by its manufacturer, is not guaranteed or endorsed by the publisher.
