# Supplementary material for: Abdominal Acupuncture as an Adjunctive Therapy for the Recovery of Motor Function After Stroke: A Systematic Review and Meta-Analysis of Randomized Controlled Trials
Source: Front Neurol. 2021 Sep 28;12:705771. doi: 10.3389/fneur.2021.705771 (PMC8505526; doi:10.3389/fneur.2021.705771)
Supplement: Supplementary file 1 [file Table_1.DOC]

Appendix 1：search in databases

**Embase**

1. 'cerebrovascular disease'/exp
2. 'brain angiopathy':ti,ab OR 'brain circulation failure':ti,ab OR 'brain vascular disease$':ti,ab OR 'cerebral small vessel disease$':ti,ab OR ('cerebral vascular':ti,ab AND (disease:ti,ab OR disorder:ti,ab OR disturbance:ti,ab OR lesion:ti,ab)) OR ((brain:ti,ab OR cerebral:ti,ab) AND vasculopathy:ti,ab) OR (cerebrovascular:ti,ab AND (damage:ti,ab OR disorder$:ti,ab OR lesion:ti,ab OR pathology:ti,ab OR syndrome:ti,ab))
3. 'basal gangli* cerebrovascular disease':ti,ab OR 'basal gangli* h$emorrhage':ti,ab OR 'cerebrovascular disease, basal gangli*':ti,ab OR 'haemorrhage, basal gangli*':ti,ab OR 'hemorrhage, basal gangli*':ti,ab OR 'h$emorrhage, putamen':ti,ab OR 'putam* h$ematoma':ti,ab OR 'putam* h$emorrhage':ti,ab OR (carotid:ti,ab AND arterial:ti,ab AND (disease$:ti,ab OR disorder$:ti,ab)) OR (carotid:ti,ab AND (arteriopathy:ti,ab OR disease:ti,ab)) OR (carotid:ti,ab AND artery:ti,ab AND (disease$:ti,ab OR disorder$:ti,ab)) OR ((cerebral:ti,ab OR intracranial:ti,ab) AND embolism:ti,ab AND thrombosis:ti,ab) OR 'carotid artery atherosclerosis':ti,ab OR ('carotid atherosclerotic':ti,ab AND (disease$:ti,ab OR plaque$:ti,ab))
4. 'artery occlusion, carotid':ti,ab OR ('carotid artery':ti,ab AND ((constriction:ti,ab OR occlusion:ti,ab OR occlusive:ti,ab) AND disease:ti,ab OR stenosis:ti,ab)) OR (carotid:ti,ab AND (obliteration:ti,ab OR occlusion:ti,ab OR stenosis:ti,ab)) OR 'arterial thrombosis, carotid':ti,ab OR 'carotid artery thrombus':ti,ab OR 'carotid thrombosis':ti,ab OR 'thrombosis, carotid artery':ti,ab OR 'artery occlusion, internal carotid':ti,ab OR 'internal carotid arterial occlusion':ti,ab OR 'internal carotid occlusion':ti,ab
5. (brain:ti,ab OR cerebral:ti,ab OR interhemispheric:ti,ab OR intracerebral:ti,ab OR intracranial:ti,ab) AND h$ematoma:ti,ab OR 'h$ematoma, brain':ti,ab OR 'bleeding, corpus callosum':ti,ab OR ((brain:ti,ab OR cerebral:ti,ab OR intracerebral:ti,ab OR intracortical:ti,ab OR intracranial:ti,ab OR periventricular:ti,ab) AND (bleeding:ti,ab OR h$emorrhage$:ti,ab OR microh$emorrhage$:ti,ab OR microbleed:ti,ab)) OR ('corpus callosum':ti,ab AND (bleeding:ti,ab OR h$emorrhage:ti,ab)) OR 'encephalorrhagia':ti,ab OR (h$emorrhage,:ti,ab AND (brain:ti,ab OR intracranial:ti,ab)) OR (h$emorrhagic:ti,ab AND (apoplexy:ti,ab OR stroke:ti,ab)) OR (h$emorrhagic:ti,ab AND stroke:ti,ab AND intracerebral:ti,ab AND bleeding:ti,ab) OR 'hematencephalon':ti,ab OR 'intracranial h$emorrhage':ti,ab OR 'posterior fossa h$emorrhage':ti,ab OR ((brain:ti,ab OR cerebral:ti,ab OR cerebrovascular:ti,ab OR cortical:ti,ab OR hemispher*:ti,ab) AND infarc*:ti,ab) OR 'infarction, brain':ti,ab
6. 'anterior cerebral artery infarction':ti,ab OR 'artery disease$, cerebral':ti,ab OR 'cerebral artery disease$':ti,ab OR (infarction,:ti,ab AND (anterior:ti,ab OR middle:ti,ab OR posterior:ti,ab) AND cerebral:ti,ab AND artery:ti,ab) OR 'intracranial arterial disease$':ti,ab OR ((middle:ti,ab OR posterior:ti,ab) AND cerebral:ti,ab AND artery:ti,ab AND infarction:ti,ab) OR 'brain stem infarctions':ti,ab OR 'brainstem infarction':ti,ab OR 'infarction, brain stem or cerebellar infarction':ti,ab
7. ('accident, cerebrovascular':ti,ab OR 'acute cerebrovascular lesion':ti,ab OR 'acute focal cerebral vasculopathy':ti,ab OR ((acute:ti,ab OR apoplectic:ti,ab OR cryptogenic:ti,ab) AND stroke:ti,ab) OR 'apoplex*':ti,ab OR 'blood flow disturbance, brain':ti,ab OR (brain:ti,ab AND (accident:ti,ab OR attack:ti,ab OR 'blood flow disturbance':ti,ab OR insul*:ti,ab OR 'isch$emic attack':ti,ab OR 'vascular accident':ti,ab)) OR (cerebral:ti,ab AND (apoplexia:ti,ab OR insul*:ti,ab OR stroke:ti,ab OR (vascular:ti,ab AND (accident:ti,ab OR insufficiency:ti,ab)))) OR 'cerebro vascular accident':ti,ab OR (cerebrovascular:ti,ab AND (arrest:ti,ab OR failure:ti,ab OR injury:ti,ab OR insufficiency:ti,ab OR insul*:ti,ab)) OR 'cerebrum vascular accident':ti,ab OR 'cva':ti,ab OR (isch$emic:ti,ab AND ('cerebral attack':ti,ab OR seizure:ti,ab)) OR 'stroke':ti,ab OR 'embolic stroke':ti,ab OR 'lacunar infarction':ti,ab OR 'stroke, lacunar':ti,ab OR 'acute isch$emic stroke':ti,ab OR (brain:ti,ab AND 'arterial insufficiency':ti,ab) OR 'circulation disorder':ti,ab OR 'brain ischaemia':ti,ab OR ('cerebral blood':ti,ab AND ('circulation disorder':ti,ab OR 'flow disorder':ti,ab)) OR (cerebral:ti,ab AND (circulation:ti,ab OR circulatory:ti,ab) AND disorder:ti,ab) OR ((cerebral:ti,ab OR cerebrovascular:ti,ab OR neural:ti,ab) AND isch$emia:ti,ab) OR 'cerebrovascular circulation disorder$':ti,ab OR 'chronic isch$emic stroke':ti,ab OR (isch$emi?:ti,ab AND cerebri:ti,ab) OR 'brain disease$':ti,ab OR encephalopathy:ti,ab OR stroke:ti,ab OR ((cerebral:ti,ab OR intracranial:ti,ab) AND vasospasm:ti,ab)) AND vasospasm,:ti,ab AND (brain:ti,ab OR cerebral:ti,ab OR intracranial:ti,ab)
8. 'anoxic isch$emic':ti,ab AND encephalopathy:ti,ab OR 'brain hypoxi? isch$emia':ti,ab OR 'brain hypoxia-isch$emia':ti,ab OR ('brain hypoxic isch$emic':ti,ab AND (damage:ti,ab OR injur*:ti,ab)) OR 'brain isch$emia hypoxia':ti,ab OR 'brain isch$emia-hypoxia':ti,ab OR 'cerebral hypoxi? isch$emia':ti,ab OR ('cerebral hypoxic isch$emic':ti,ab AND (damage:ti,ab OR injur*:ti,ab)) OR 'cerebral isch$emia hypoxia':ti,ab OR 'hie (hypoxic isch$emic encephalopathy)':ti,ab OR 'hypoxia-isch$emia, brain':ti,ab OR ('hypoxic isch$emic':ti,ab AND (brain:ti,ab OR cerebral:ti,ab) AND (damage:ti,ab OR injur*:ti,ab)) OR 'hypoxic isch$emic encephalopathy':ti,ab
9. 'brain transient isch$emic attack':ti,ab OR 'cerebral isch$emia, transient':ti,ab OR 'circulatory epilepsy':ti,ab OR 'epilepsy circulatory':ti,ab OR 'isch$emic attack':ti,ab OR 'tia':ti,ab OR (transient:ti,ab AND (brain:ti,ab OR cerebral:ti,ab) AND isch$emia:ti,ab) OR ('transient isch$emic':ti,ab AND (attack:ti,ab OR seizure:ti,ab)) OR 'brain blood vessel malformation':ti,ab OR ((brain:ti,ab OR cerebral:ti,ab OR cerebrum:ti,ab) AND vascular:ti,ab AND (anomaly:ti,ab OR malformation:ti,ab)) OR 'cerebrovascular anomaly':ti,ab OR 'vascular malformation, cerebrum':ti,ab
10. 'aneurysm, intracranial':ti,ab OR 'arteriovenous aneurysm, brain':ti,ab OR 'arteriovenous cerebral aneurysm':ti,ab OR 'arteriovenous fistula, brain':ti,ab OR 'brain aneurysm, arteriovenous':ti,ab OR ('brain arteriovenous':ti,ab AND (aneurysm:ti,ab OR fistula:ti,ab OR shunt:ti,ab)) OR 'cerebral aneurysm, arteriovenous':ti,ab OR ('cerebral arteriovenous':ti,ab AND (fistula:ti,ab OR malformation$:ti,ab)) OR 'intracranial arteriovenous malformation$':ti,ab
11. 'brain artery':ti,ab AND (obstruction:ti,ab OR occlusion:ti,ab OR thrombosis:ti,ab) OR (brain:ti,ab AND (thromboembolism:ti,ab OR thrombosis:ti,ab)) OR 'brain vascular obstruction':ti,ab OR ('cerebral artery':ti,ab AND (occlusion:ti,ab OR thrombosis:ti,ab)) OR 'cerebral thrombosis':ti,ab OR 'cerebrovascular disease, occlusive':ti,ab OR (cerebrovascular:ti,ab AND (obliteration:ti,ab OR obstruction:ti,ab OR occlusion:ti,ab)) OR 'cerebrovascular occlusi* disease':ti,ab OR 'cerebrovascular thrombosis':ti,ab OR 'intracranial artery thrombosis':ti,ab OR 'intracranial thrombosis':ti,ab OR 'thromboembolism, brain':ti,ab OR (thrombosis:ti,ab AND (cerebri:ti,ab OR intracranial:ti,ab)) OR 'thrombosis, brain artery':ti,ab
12. medial:ti,ab AND (brain:ti,ab OR cerebral:ti,ab) AND 'artery occlusion':ti,ab OR 'middle cerebral artery stenosis':ti,ab OR 'arteria vertebralis occlusion':ti,ab OR ('vertebral artery':ti,ab AND (obstruction:ti,ab OR occlusion:ti,ab)) OR 'vertebral occlusion':ti,ab OR ('lateral medullary':ti,ab AND (disease:ti,ab OR syndrome:ti,ab)) OR 'wallenberg syndrome':ti,ab
13. #1 OR #2 OR #3 OR #4 OR #5 OR #6 OR #7 OR #8 OR #9 OR #10 OR #11 OR #12
14. 'motor activity'/exp OR 'motor dysfunction'/exp
15. 'activity, motor':ti,ab OR 'disability, motor':ti,ab OR (motor:ti,ab AND (disability:ti,ab OR disfunction:ti,ab OR disorder$:ti,ab OR disturbance:ti,ab OR impairment:ti,ab)) OR 'movement disorder$':ti,ab OR 'stereotypic movement disorder':ti,ab
16. 'ataxie':ti,ab OR 'contracture$':ti,ab OR ((abnormal:ti,ab OR disordered:ti,ab) AND coordination:ti,ab) OR (extrapyramidal:ti,ab AND (phenomenon:ti,ab OR 'side effect':ti,ab OR sign:ti,ab)) OR 'symptom, extrapyramidal':ti,ab OR 'abnormal gait':ti,ab OR 'gait deviation$':ti,ab OR 'chair bound':ti,ab OR 'chairbound':ti,ab OR 'chairfast':ti,ab OR 'wheelchair bound':ti,ab OR 'wheelchair bounded':ti,ab OR 'hypertonia':ti,ab OR 'hypertonus':ti,ab OR 'muscle hypertonicity':ti,ab OR 'muscle hypertonus':ti,ab OR 'muscular hypertonia':ti,ab OR 'hypotonia':ti,ab OR 'hypotonia musculorum':ti,ab OR 'hypotonicity':ti,ab OR 'hypotonus':ti,ab OR 'muscle hypotonus':ti,ab OR 'muscle hypotony':ti,ab OR 'muscular hypotonia':ti,ab OR 'involuntary muscle contraction':ti,ab OR 'muscular spasm':ti,ab OR 'myospasia':ti,ab OR 'myospasm':ti,ab OR 'myospasmia':ti,ab OR 'spasm':ti,ab OR 'spasm, muscle':ti,ab OR ((muscle:ti,ab OR muscular:ti,ab) AND (strength:ti,ab AND loss:ti,ab OR weaken*:ti,ab OR insufficiency:ti,ab)) OR 'neuromuscular fatigue':ti,ab OR 'weakness, muscle':ti,ab OR (pyramidal:ti,ab AND (disorder$:ti,ab OR lesion:ti,ab OR symptom:ti,ab OR syndrome:ti,ab OR (system:ti,ab AND lesion:ti,ab) OR (tract:ti,ab AND (disorder:ti,ab OR lesion:ti,ab OR sign:ti,ab)))) OR 'pyramidalis syndrome':ti,ab OR 'syndrome, pyramidal':ti,ab
17. 'static tremor':ti,ab OR 'tremor induction':ti,ab OR 'tremor, static':ti,ab OR 'tremorogenesis':ti,ab OR 'tremulousness':ti,ab OR 'ambulat* difficult*':ti,ab OR 'dependent ambulation':ti,ab OR 'difficulty walking':ti,ab OR (mobility:ti,ab AND (disabilit*:ti,ab OR limitation$:ti,ab)) OR 'walking difficult*':ti,ab OR 'hemiparalysis':ti,ab OR 'hemiplegic':ti,ab OR 'hemiplegy':ti,ab
18. #14 OR #15 OR #16 OR #17
19. 'abdominal acupuncture':ti,ab OR 'ventro acupuncture':ti,ab OR 'abdomen needle':ti,ab OR 'abdomen acupuncture':ti,ab
20. 'randomized controlled trial'/exp OR 'clinical study'/exp OR 'clinical trial'/exp OR 'controlled clinical trial'/exp OR 'case control study'/exp OR 'comparative study'/exp OR 'equivalence trial'/exp OR 'pragmatic clinical trial':ab,ti OR 'randomized controlled trial (topic)'/exp OR 'clinical trials (topic)' OR 'controlled clinical trial (topic)'/exp OR 'equivalence trial (topic)'/exp OR 'clinical trial$, randomized':ab,ti OR 'trial$, randomized clinical':ab,ti OR 'comparative stud*':ab,ti OR 'practical clinical trial$':ab,ti OR 'clinical trial$, practical':ab,ti OR 'trial$, practical clinical':ab,ti OR 'pragmatic trial$':ab,ti OR 'trial$, pragmatic':ab,ti OR 'pragmatic clinical trial$':ab,ti OR 'random allocation':ab,ti OR 'allocation,random':ab,ti OR 'randomization'/exp OR 'random*':ab,ti OR 'placebo':ab,ti OR 'trial$':ab,ti OR 'case-control':ab,ti OR 'case control':ab,ti OR (('case comparison':ab,ti OR 'case compeer':ab,ti OR 'case referrent':ab,ti OR 'case base':ab,ti) AND stud*:ab,ti) OR (stud*,:ab,ti AND ('case comparison':ab,ti OR 'case compeer':ab,ti OR 'case referrent':ab,ti OR 'case base':ab,ti)) OR 'comparative study':ab,ti
21. 'animal experimentation'/exp OR 'animal':ab,ti OR 'mouse':ab,ti OR 'mice':ab,ti OR 'rats':ab,ti
22. #20 NOT #21
23. #13 AND #18 AND #19 AND #22 AND ([article]/lim OR [article in press]/lim) AND [humans]/lim AND [clinical study]/lim AND ([controlled clinical trial]/lim OR [randomized controlled trial]/lim) AND [<1966-2020]/py

**Cochrane Library**

1. MeSH descriptor: [Cerebrovascular Disorders] explode all trees
2. (((Cerebrovascular OR Intracranial) (Disease? OR Disorder?)) OR Brain Vascular Disorder? OR Vascular Disorder?, Brain OR Vascular Disorder?, Intracranial OR Vascular Disease?, Intracranial OR Intracranial Vascular Disease? OR Disease?, Cerebrovascular OR Vascular Disorder?, Intracranial OR (Cerebrovascular (Insufficienc* OR Occlusion?)) OR Insufficienc*, Cerebrovascular OR Occlusion?, Cerebrovascular):ti,ab,kw (Word variations have been searched)
3. (Arter* Disease?, Carotid OR Arter* Disorder?, Carotid OR Disorder?, Carotid Artery OR Carotid Arter* Disorder? OR Carotid Arter* Disease? OR Arterial Disease?, External Carotid OR External Carotid Artery Disease? OR Atherosclerotic Disease?, Carotid OR Carotid Atherosclerotic Disease? OR Carotid Atheroscleros* OR Arter* Disease?, Common Carotid OR Internal Carotid Arter* Disease? OR Arter* Disease?, Internal Carotid):ti,ab,kw (Word variations have been searched)
4. ((Arter* Brain (Disorder? OR Disease?)) OR Arter* Disease?, Brain OR ((Brain OR Intracranial) Arter* (Disorder? OR Disease?)) OR Brain Disorder?, Arter* OR Brain Disease?, Arter* OR Arter* Brain Disease OR Arter* Disease?, Intracranial OR Arter* Disorder?, Intracranial):ti,ab,kw (Word variations have been searched) 155650
5. (((Posterior Fossa OR Cerebrum OR Brain OR Intracranial OR Cerebral OR Parenchymal OR Intracerebral OR Ganglionic OR Putamen) Hemorrhage?) OR Hemorrhage?, Posterior Fossa OR Hemorrhage?, Cerebrum OR Hemorrhage?, Cerebral OR Hemorrhage?, Brain OR Hemorrhage?, Intracranial OR Hemorrhage?, Intracerebral OR Hemorrhage?, Cerebrum OR Hemorrhage?, Basal Gangli* OR Hematoma?, Basal Gangli* OR Hemorrhage, Putam* OR Putaminal Hematoma?):ti,ab,kw (Word variations have been searched)
6. (((Cerebral OR Brain) Ischemia?) OR Ischemia?, Cerebral OR Ischemic Encephalopath* OR Encephalopath*, Ischemic OR Ischemia?, Brain OR Brain Infarct* OR Infarct*, Brain OR Infarction?, Posterior Circulation, Brain OR Anterior Cerebral Circulation Infarction? OR Infarction?, Anterior Cerebral Circulation OR Infarction?, Anterior Circulation, Brain):ti,ab,kw (Word variations have been searched)
7. (((Benedict OR Claude OR Foville OR Weber OR Millard-Gublar OR Millard-Gublar OR Top of the Basilar OR Dorsolateral Medullary OR Vieseaux-Wallenberg OR Lateral Bulbar OR Wallenberg OR Lateral Medullary OR Posterior Inferior Cerebellar Artery OR Vieseaux Wallenberg OR Wallenberg's OR Wallenbergs) Syndrome?) OR Infarction?, Brainstem OR Brainstem Infarction? OR Stem Infarct*, Brain OR Infarct*, Brain Stem OR Brain Stem Infarct* OR Brainstem Stroke OR Stroke, Brainstem OR Syndrome, Millard-Gublar OR Syndrome?, Lateral Medullary OR Syndrom?, Vieseaux-Wallenberg OR Syndrome?, Wallenberg OR Medullary Syndrome?, Dorsolateral):ti,ab,kw (Word variations have been searched)
8. (((Cerebral OR Brain) (Ischemia Hypoxia? OR Hypoxia-Ischemia? OR Ischemia-Hypoxia? OR Hypoxia Ischemia? OR Anoxia-Ischemia? OR Ischemia-Anoxia? OR Anoxia Ischemia? OR Ischemia Anoxia?)) OR Ischemia-Hypoxia?, Cerebral OR Hypoxia Ischemia?, Cerebral OR Ischemia Hypoxia?, Cerebral OR Hypoxia-Ischemia?, Cerebral OR Ischemia-Hypoxia?, Brain OR Ischemia Hypoxia?, Brain OR Hypoxia-Ischemias, Brain OR Hypoxia Ischemia, Brain OR Ischemia Anoxia?, Cerebral OR Anoxia-Ischemia?, Cerebral OR Anoxia Ischemia?, Cerebral OR Ischemia-Anoxia, Cerebral OR Anoxia-Ischemia?, Brain OR Ischemia-Anoxia?, Brain OR Anoxia Ischemia?, Brain OR Ischemia Anoxia?, Brain OR ((Anoxic-Ischemic OR Anoxic Ischemic OR Hypoxic-Ischemic OR Ischemic-Hypoxic OR Hypoxic Ischemic OR Ischemic Hypoxic) Encephalopath*) OR Encephalopath*, Anoxic Ischemic OR Encephalopath*, Anoxic-Ischemic OR Encephalopath*, Ischemic-Hypoxic OR Encephalopath*, Hypoxic Ischemic OR Encephalopath*, Hypoxic-Ischemic):ti,ab,kw (Word variations have been searched)
9. (((Vertebral OR Basilar) Artery (Insufficienc* OR Ischemia? OR Stenos?s)) OR Insufficienc*, Basilar Artery OR Insufficienc*, Basilar OR Artery Ischemia?, Basilar OR Ischemia?, Basilar Artery OR Basilar Insufficienc* OR Stenos?s, Basilar Artery OR Artery Stenos?s, Basilar OR Insufficienc*, Vertebral Artery OR Artery Ischemia?, Vertebral OR Artery Insufficienc*, Vertebral OR Ischemia?, Vertebral Artery OR ((Vertebro-Basilar OR Vertebrobasilar OR Vertebro Basilar) (Ischemia? OR Insufficienc* OR Dolichoectasia?)) OR Ischemia?, Vertebro-Basilar OR Insufficienc?, Vertebro-Basilar OR Insufficienc?, Vertebrobasilar OR Ischemia?, Vertebrobasilar OR Ischemia, Vertebrobasilar OR Ischemia?, Vertebro-Basilar OR Dolichoectasia?, Vertebrobasilar OR Vertebrobasilar Dolichoectasia? OR Stenos?s, Vertebral Artery OR Artery Stenos?s, Vertebral):ti,ab,kw (Word variations have been searched) 53067
10. ("Cerebral Embolism and Thrombosis" OR "Embolism and Thrombosis, Brain" OR "Brain Embolism and Thrombosis"):ti,ab,kw (Word variations have been searched)
11. (Apoplexy OR ((Acute OR Cerebral OR Cerebrovascular) Stroke?) OR ((Cerebrovascular OR Brain Vascular) Accident?) OR Cerebrovascular Apoplexy OR Cerebrovascular Accident?, Acute OR Vascular Accident?, Brain OR Stroke?, Cerebrovascular OR Stroke?, Acute OR Stroke?, Cerebral):ti,ab,kw (Word variations have been searched)
12. #1 OR #2 OR #3 OR #4 OR #5 OR #6 OR #7 OR #8 OR #9 OR #10 OR #11
13. MeSH descriptor: [Motor Activity] explode all trees
14. MeSH descriptor: [Motor Disorders] explode all trees
15. (Activit*, Motor OR Motor Activit* OR hemiplegic OR motor dysfunction):ti,ab,kw (Word variations have been searched)
16. #13 OR #14 OR #15
17. (abdominal acupuncture OR ventro acupuncture OR abdomen needle OR abdomen acupuncture):ti,ab,kw (Word variations have been searched)
18. MeSH descriptor: [Controlled Clinical Trial] explode all trees
19. (Superiority Trial? OR Non-Inferiority Trial? OR Noninferiority Trial?? OR Equivalence Clinical Trial?):ti,ab,kw (Word variations have been searched)
20. (randomized control):ti,ab,kw (Word variations have been searched)
21. #18 OR #19 OR #20
22. #12 AND #16 AND #17 AND #21 with Cochrane Library publication date Between Jan 1900 and Dec 2020

**PubMed**

1. cerebrovascular disorders[mh]
2. Cerebrovascular Disease*[tiab] OR Intracranial Disorder*[tiab] OR Cerebrovascular Disorder*[tiab] OR Intracranial Disease*[tiab] OR Brain Vascular Disorder*[tiab] OR Vascular Disorder*, Brain[tiab] OR Vascular Disorder*, Intracranial[tiab] OR Vascular Disease*, Intracranial[tiab] OR Intracranial Vascular Disease*[tiab] OR Disease*, Cerebrovascular[tiab] OR Vascular Disorder*, Intracranial[tiab] OR Cerebrovascular Insufficienc*[tiab] OR Cerebrovascular Occlusion*[tiab] OR Insufficienc*, Cerebrovascular[tiab] OR Occlusion*, Cerebrovascular[tiab]
3. Arter* Disease*, Carotid[tiab] OR Arter* Disorder*, Carotid[tiab] OR Disorder*, Carotid Artery[tiab] OR Carotid Arter* Disorder*[tiab] OR Carotid Arter* Disease*[tiab] OR Arterial Disease*, External Carotid[tiab] OR External Carotid Artery Disease*[tiab] OR Atherosclerotic Disease*, Carotid[tiab] OR Carotid Atherosclerotic Disease*[tiab] OR Carotid Atheroscleros*[tiab] OR Arter* Disease*, Common Carotid[tiab] OR Internal Carotid Arter* Disease*[tiab] OR Arter* Disease*, Internal Carotid[tiab]
4. Arter* Brain Disorder*[tiab] OR Arter* Brain Disease*[tiab] OR Arter* Disease*, Brain[tiab] OR Brain Arter* Disorder*[tiab] OR Intracranial Arter* Disease*[tiab] OR Brain Arter* Disease*[tiab] OR Intracranial Arter* Disorder*[tiab] OR Brain Disorder*, Arter*[tiab] OR Brain Disease*, Arter*[tiab] OR Arter* Brain Disease[tiab] OR Arter* Disease*, Intracranial[tiab] OR Arter* Disorder*, Intracranial[tiab]
5. Posterior Fossa Hemorrhage*[tiab] OR Cerebrum Hemorrhage*[tiab] OR Brain Hemorrhage*[tiab] OR Intracranial Hemorrhage*[tiab] OR Cerebral Hemorrhage*[tiab] OR Parenchymal Hemorrhage*[tiab] OR Intracerebral Hemorrhage*[tiab] OR Ganglionic Hemorrhage*[tiab] OR Putamen Hemorrhage*[tiab] OR Hemorrhage*, Posterior Fossa[tiab] OR Hemorrhage*, Cerebrum[tiab] OR Hemorrhage*, Cerebral[tiab] OR Hemorrhage*, Brain[tiab] OR Hemorrhage*, Intracranial[tiab] OR Hemorrhage*, Intracerebral[tiab] OR Hemorrhage*, Cerebrum[tiab] OR Hemorrhage*, Basal Gangli*[tiab] OR Hematoma*, Basal Gangli*[tiab] OR Hemorrhage, Putam*[tiab] OR Putaminal Hematoma*[tiab]
6. Cerebral Ischemia*[tiab] OR Brain Ischemia*[tiab] OR Ischemia*, Cerebral[tiab] OR Ischemic Encephalopath*[tiab] OR Encephalopath*, Ischemic[tiab] OR Ischemia*, Brain[tiab] OR Brain Infarct*[tiab] OR Infarct*, Brain[tiab] OR Infarction*, Posterior Circulation, Brain[tiab] OR Anterior Cerebral Circulation Infarction*[tiab] OR Infarction*, Anterior Cerebral Circulation[tiab] OR Infarction*, Anterior Circulation, Brain[tiab]
7. Benedict Syndrome*[tiab] OR Claude Syndrome*[tiab] OR Foville Syndrome*[tiab] OR Weber Syndrome*[tiab] OR Millard-Gublar Syndrome*[tiab] OR Millard-Gublar Syndrome*[tiab] OR Top of the Basilar Syndrome*[tiab] OR Dorsolateral Medullary Syndrome*[tiab] OR Lateral Bulbar[tiab] OR Wallenberg Syndrome*[tiab] OR Lateral Medullary Syndrome*[tiab] OR Posterior Inferior Cerebellar Artery Syndrome*[tiab] OR Wallenberg's Syndrome*[tiab] OR Wallenbergs Syndrome*[tiab] OR Infarction*, Brainstem[tiab] OR Brainstem Infarction*[tiab] OR Stem Infarct*, Brain[tiab] OR Infarct*, Brain Stem[tiab] OR Brain Stem Infarct*[tiab] OR Brainstem Stroke[tiab] OR Stroke, Brainstem[tiab] OR Syndrome, Millard-Gublar[tiab] OR Syndrome*, Lateral Medullary[tiab] OR Syndrome*, Wallenberg[tiab] OR Medullary Syndrome*, Dorsolateral[tiab]
8. Cerebral Ischemia Hypoxia*[tiab] OR Cerebral Hypoxia-Ischemia*[tiab] OR Cerebral Ischemia-Hypoxia*[tiab] OR Cerebral Hypoxia Ischemia*[tiab] OR Cerebral Anoxia-Ischemia*[tiab] OR Cerebral Ischemia-Anoxia*[tiab] OR Cerebral Anoxia Ischemia*[tiab] OR Cerebral Ischemia Anoxia*[tiab] OR Brain Ischemia Hypoxia*[tiab] OR Brain Hypoxia-Ischemia*[tiab] OR Brain Ischemia-Hypoxia*[tiab] OR Brain Hypoxia Ischemia*[tiab] OR Brain Anoxia-Ischemia*[tiab] OR Brain Ischemia-Anoxia*[tiab] OR Brain Anoxia Ischemia*[tiab] OR Brain Ischemia Anoxia*[tiab] OR Ischemia-Hypoxia*, Cerebral[tiab] OR Hypoxia Ischemia*, Cerebral[tiab] OR Ischemia Hypoxia*, Cerebral[tiab] OR Hypoxia-Ischemia*, Cerebral[tiab] OR Ischemia-Hypoxia*, Brain[tiab] OR Ischemia Hypoxia*, Brain[tiab] OR Hypoxia-Ischemias, Brain[tiab] OR Hypoxia Ischemia, Brain[tiab] OR Ischemia Anoxia*, Cerebral[tiab] OR Anoxia-Ischemia*, Cerebral[tiab] OR Anoxia Ischemia*, Cerebral[tiab] OR Ischemia-Anoxia, Cerebral[tiab] OR Anoxia-Ischemia*, Brain[tiab] OR Ischemia-Anoxia*, Brain[tiab] OR Anoxia Ischemia*, Brain[tiab] OR Ischemia Anoxia*, Brain[tiab] OR Anoxic-Ischemic Encephalopath*[tiab] OR Anoxic Ischemic Encephalopath*[tiab] OR Hypoxic-Ischemic Encephalopath*[tiab] OR Ischemic-Hypoxic Encephalopath*[tiab] OR Hypoxic Ischemic Encephalopath*[tiab] OR Ischemic Hypoxic Encephalopath*[tiab] OR Encephalopath*, Anoxic Ischemic[tiab] OR Encephalopath*, Anoxic-Ischemic[tiab] OR Encephalopath*, Ischemic-Hypoxic[tiab] OR Encephalopath*, Hypoxic Ischemic[tiab] OR Encephalopath*, Hypoxic-Ischemic[tiab]
9. Transient Ischemic Attack*[tiab] OR Transient Cerebral Ischemia*[tiab] OR Attack*, Transient Ischemic[tiab] OR Ischemic Attack*, Transient[tiab] OR Cerebral Ischemia*, Transient[tiab] OR Ischemia*, Transient Cerebral[tiab] OR Transient Brainstem Ischemia*[tiab] OR Brainstem Ischemia*, Transient[tiab] OR Ischemia*, Transient Brainstem[tiab] OR Brain Stem Ischemia, Transient[tiab]
10. Vertebral Artery Insufficienc*[tiab] OR Vertebral Artery Ischemia*[tiab] OR Vertebral Artery Stenos*[tiab] OR Basilar Artery Insufficienc*[tiab] OR Basilar Artery Ischemia*[tiab] OR Basilar Artery Stenos*[tiab] OR Insufficienc*, Basilar Artery[tiab] OR Insufficienc*, Basilar[tiab] OR Artery Ischemia*, Basilar[tiab] OR Ischemia*, Basilar Artery[tiab] OR Basilar Insufficienc*[tiab] OR Stenos*, Basilar Artery[tiab] OR Artery Stenos*, Basilar[tiab] OR Insufficienc*, Vertebral Artery[tiab] OR Artery Ischemia*, Vertebral[tiab] OR Artery Insufficienc*, Vertebral[tiab] OR Ischemia*, Vertebral Artery[tiab] OR Vertebro-Basilar Ischemia*[tiab] OR Vertebro-Basilar Insufficienc*[tiab] OR Vertebro-Basilar Dolichoectasia*[tiab] OR Vertebrobasilar Ischemia*[tiab] OR Vertebrobasilar Insufficienc*[tiab] OR Vertebrobasilar Dolichoectasia*[tiab] OR Vertebro Basilar Ischemia*[tiab] OR Vertebro Basilar Insufficienc*[tiab] OR Vertebro Basilar Dolichoectasia*[tiab] OR Ischemia*, Vertebro-Basilar[tiab] OR Insufficienc*, Vertebro-Basilar[tiab] OR Insufficienc*, Vertebrobasilar[tiab] OR Ischemia*, Vertebrobasilar[tiab] OR Ischemia, Vertebrobasilar[tiab] OR Ischemia*, Vertebro-Basilar[tiab] OR Dolichoectasia*, Vertebrobasilar[tiab] OR Vertebrobasilar Dolichoectasia*[tiab] OR Stenos*, Vertebral Artery[tiab] OR Artery Stenos*, Vertebral[tiab] OR "Cerebral Embolism and Thrombosis"[tiab]
11. Apoplexy[tiab] OR Acute Stroke*[tiab] OR Cerebral Stroke*[tiab] OR Cerebrovascular Stroke*[tiab] OR Cerebrovascular Accident*[tiab] OR Brain Vascular Accident*[tiab] OR Cerebrovascular Apoplexy[tiab] OR Cerebrovascular Accident*, Acute[tiab] OR Vascular Accident*, Brain[tiab] OR Stroke*, Cerebrovascular[tiab] OR Stroke*, Acute[tiab] OR Stroke*, Cerebral[tiab]
12. #1 OR #2 OR #3 OR #4 OR #5 OR #6 OR #7 OR #8 OR #9 OR #10 OR #11
13. motor activity[mh] OR motor disorder[mh]
14. Activit*, Motor[tiab] OR Motor Activit*[tiab] OR hemiplegic[tiab] OR motor dysfunction[tiab]
15. #13 OR #14
16. abdominal acupuncture[tiab] OR ventro acupuncture[tiab] OR abdomen needle[tiab] OR abdomen acupuncture[tiab]
17. randomized controlled trial[pt] OR Randomized Controlled Trials as Topic[mh:noexp]
18. Superiority Trial*[tiab] OR Non-Inferiority Trial*[tiab] OR Noninferiority Trial**[tiab] OR Equivalence Clinical Trial*[tiab] OR randomized control[tiab]
19. #17 OR #18
20. #12 AND #15 AND #16 AND #19
21. #20 Filters: from 1900/1/1 - 2020/12/31

**CNKI**

( ( ( ( 主题%= xls(中风,'SYS_XL_SYNONYM_DICT')+xls(脑卒中,'SYS_XL_SYNONYM_DICT')+xls(脑血管病,'SYS_XL_SYNONYM_DICT')+xls(脑血管意外,'SYS_XL_SYNONYM_DICT')+xls(脑梗死,'SYS_XL_SYNONYM_DICT')+xls(脑梗塞,'SYS_XL_SYNONYM_DICT')+xls(脑血栓,'SYS_XL_SYNONYM_DICT')+xls(脑栓塞,'SYS_XL_SYNONYM_DICT')+xls(脑出血,'SYS_XL_SYNONYM_DICT')+xls(脑溢血,'SYS_XL_SYNONYM_DICT') or 题名%= xls(中风,'SYS_XL_SYNONYM_DICT')+xls(脑卒中,'SYS_XL_SYNONYM_DICT')+xls(脑血管病,'SYS_XL_SYNONYM_DICT')+xls(脑血管意外,'SYS_XL_SYNONYM_DICT')+xls(脑梗死,'SYS_XL_SYNONYM_DICT')+xls(脑梗塞,'SYS_XL_SYNONYM_DICT')+xls(脑血栓,'SYS_XL_SYNONYM_DICT')+xls(脑栓塞,'SYS_XL_SYNONYM_DICT')+xls(脑出血,'SYS_XL_SYNONYM_DICT')+xls(脑溢血,'SYS_XL_SYNONYM_DICT') or title= xls(中风,'SYS_XL_SYNONYM_DICT')+xls(脑卒中,'SYS_XL_SYNONYM_DICT')+xls(脑血管病,'SYS_XL_SYNONYM_DICT')+xls(脑血管意外,'SYS_XL_SYNONYM_DICT')+xls(脑梗死,'SYS_XL_SYNONYM_DICT')+xls(脑梗塞,'SYS_XL_SYNONYM_DICT')+xls(脑血栓,'SYS_XL_SYNONYM_DICT')+xls(脑栓塞,'SYS_XL_SYNONYM_DICT')+xls(脑出血,'SYS_XL_SYNONYM_DICT')+xls(脑溢血,'SYS_XL_SYNONYM_DICT') or v_subject= xls(中风,'SYS_XL_SYNONYM_DICT')+xls(脑卒中,'SYS_XL_SYNONYM_DICT')+xls(脑血管病,'SYS_XL_SYNONYM_DICT')+xls(脑血管意外,'SYS_XL_SYNONYM_DICT')+xls(脑梗死,'SYS_XL_SYNONYM_DICT')+xls(脑梗塞,'SYS_XL_SYNONYM_DICT')+xls(脑血栓,'SYS_XL_SYNONYM_DICT')+xls(脑栓塞,'SYS_XL_SYNONYM_DICT')+xls(脑出血,'SYS_XL_SYNONYM_DICT')+xls(脑溢血,'SYS_XL_SYNONYM_DICT') ) AND ( 主题%= xls(运动障碍,'SYS_XL_SYNONYM_DICT')+xls(运动功能,'SYS_XL_SYNONYM_DICT')+xls(偏瘫,'SYS_XL_SYNONYM_DICT') or 题名%= xls(运动障碍,'SYS_XL_SYNONYM_DICT')+xls(运动功能,'SYS_XL_SYNONYM_DICT')+xls(偏瘫,'SYS_XL_SYNONYM_DICT') or title= xls(运动障碍,'SYS_XL_SYNONYM_DICT')+xls(运动功能,'SYS_XL_SYNONYM_DICT')+xls(偏瘫,'SYS_XL_SYNONYM_DICT') or v_subject= xls(运动障碍,'SYS_XL_SYNONYM_DICT')+xls(运动功能,'SYS_XL_SYNONYM_DICT')+xls(偏瘫,'SYS_XL_SYNONYM_DICT') ) ) AND ( 主题%= xls(腹针,'SYS_XL_SYNONYM_DICT')+xls(腹部针刺,'SYS_XL_SYNONYM_DICT')+xls(腹部针灸,'SYS_XL_SYNONYM_DICT') or 题名%= xls(腹针,'SYS_XL_SYNONYM_DICT')+xls(腹部针刺,'SYS_XL_SYNONYM_DICT')+xls(腹部针灸,'SYS_XL_SYNONYM_DICT') or title= xls(腹针,'SYS_XL_SYNONYM_DICT')+xls(腹部针刺,'SYS_XL_SYNONYM_DICT')+xls(腹部针灸,'SYS_XL_SYNONYM_DICT') or v_subject= xls(腹针,'SYS_XL_SYNONYM_DICT')+xls(腹部针刺,'SYS_XL_SYNONYM_DICT')+xls(腹部针灸,'SYS_XL_SYNONYM_DICT') ) ) AND ( 主题%= xls(随机对照,'SYS_XL_SYNONYM_DICT')+xls(对照,'SYS_XL_SYNONYM_DICT')+xls(随机,'SYS_XL_SYNONYM_DICT') or 题名%= xls(随机对照,'SYS_XL_SYNONYM_DICT')+xls(对照,'SYS_XL_SYNONYM_DICT')+xls(随机,'SYS_XL_SYNONYM_DICT') or title= xls(随机对照,'SYS_XL_SYNONYM_DICT')+xls(对照,'SYS_XL_SYNONYM_DICT')+xls(随机,'SYS_XL_SYNONYM_DICT') or v_subject= xls(随机对照,'SYS_XL_SYNONYM_DICT')+xls(对照,'SYS_XL_SYNONYM_DICT')+xls(随机,'SYS_XL_SYNONYM_DICT') ) ) AND ( 发表时间 Between('1915-01-01','2020-12-31') )

**CBM**

1. (( "中风"[加权:扩展] OR "脑卒中"[加权:扩展] OR "脑血管病"[加权:扩展] OR "脑血管意外"[加权:扩展] OR "脑梗死"[加权:扩展] OR "脑梗塞"[加权:扩展] OR "脑血栓"[加权:扩展] OR "脑栓塞"[加权:扩展] OR "脑出血"[加权:扩展] OR "脑溢血"[加权:扩展])) AND ( 多中心研究[文献类型] OR 临床试验[文献类型] OR 随机对照试验[文献类型]) AND ( 人类[特征词]) AND 1900-2020[日期]
2. (( "运动障碍"[加权:扩展] OR "运动功能"[加权:扩展] OR "偏瘫"[加权:扩展])) AND ( 多中心研究[文献类型] OR 随机对照试验[文献类型] OR 临床试验[文献类型]) AND ( 人类[特征词]) AND 1900-2020[日期]
3. (( "腹针"[常用字段:智能] OR "腹部针刺"[常用字段:智能] OR "腹部针灸"[常用字段:智能])) AND ( 人类[特征词])
4. ((#1) AND (#2) AND (#3)) AND ( 人类[特征词]) AND 1900-2020[日期]

**VIP**

(((((((((((((题名或关键词=中风 OR 题名或关键词=脑卒中) OR 题名或关键词=脑血管病) OR 题名或关键词=脑血管意外) OR 题名或关键词=脑梗死) OR 题名或关键词=脑梗塞) OR 题名或关键词=脑血栓) OR 题名或关键词=脑栓塞) OR 题名或关键词=脑出血) OR 题名或关键词=脑溢血) AND ((题名或关键词=运动障碍 OR 题名或关键词=运动功能) OR 题名或关键词=偏瘫))) AND ((题名或关键词=腹针 OR 题名或关键词=腹部针刺) OR 题名或关键词=腹部针灸))) AND (years:[1900 TO 2020])

**WAN FANG**

(主题:(中风 or 脑卒中 or 脑血管病 or 脑血管意外 or 脑梗死 or 脑梗塞 or 脑血栓 or 脑栓塞 or 脑出血 or 脑溢血) and 主题:(运动障碍 or 运动功能 or 偏瘫) and 主题:(腹针 or 腹部针刺 or 腹部针灸) and 主题:(随机对照 or 对照 or 随机)) and Date:1900-2020
